# Structure and selectivity of a glutamate-specific TAXI TRAP binding protein from Vibrio cholerae

**DOI:** 10.1085/jgp.202413584

**Published:** 2024-11-18

**Authors:** Joseph F.S. Davies, Andrew Daab, Nicholas Massouh, Corey Kirkland, Bernadette Strongitharm, Andrew Leech, Marta Farré, Gavin H. Thomas, Christopher Mulligan

**Affiliations:** 1 School of Biosciences, Division of Natural Sciences, University of Kent, Canterbury, UK; 2 Department of Biology and York Biomedical Research Institute (YBRI), University of York, York, UK; 3 Technology Facility, Department of Biology, University of York, York, UK

## Abstract

Tripartite ATP-independent periplasmic (TRAP) transporters are widespread in prokaryotes and are responsible for the transport of a variety of different ligands, primarily organic acids. TRAP transporters can be divided into two subclasses; DctP-type and TAXI type, which share the same overall architecture and substrate-binding protein requirement. DctP-type transporters are very well studied and have been shown to transport a range of compounds including dicarboxylates, keto acids, and sugar acids. However, TAXI-type transporters are relatively poorly understood. To address this gap in our understanding, we have structurally and biochemically characterized VC0430 from *Vibrio cholerae*. We show it is a monomeric, high affinity glutamate-binding protein, which we thus rename VcGluP. VcGluP is stereoselective, binding the L-isomer preferentially, and can also bind L-glutamine and L-pyroglutamate with lower affinity. Structural characterization of ligand-bound VcGluP revealed details of its binding site and biophysical characterization of binding site mutants revealed the substrate binding determinants, which differ substantially from those of DctP-type TRAPs. Finally, we have analyzed the interaction between VcGluP and its cognate membrane component, VcGluQM (formerly VC0429) in silico, revealing an architecture hitherto unseen. To our knowledge, this is the first transporter in *V. cholerae* to be identified as specific to glutamate, which plays a key role in the osmoadaptation of *V. cholerae*, making this transporter a potential therapeutic target.

## Teaser

Bacterial TAXI-TRAP transporters are widespread but poorly understood. Davies et al. characterize a TAXI from *Vibrio cholerae*, revealing its glutamate specificity, binding determinants, and unusual membrane component. As glutamate is a key metabolite in pathogens, glutamate transporters are potential therapeutic targets.

## Introduction

The ability to take up extracellular solutes is essential for bacteria to thrive in an environmental niche or adapt to changing conditions. Prokaryotes have developed many transport systems that vary based on the substrates they transport, the energy source they use, the rate of transport required, and the affinity for their substrate. Many transporter families are secondary active transporters and utilize electrochemical gradients across the membrane (usually Na^+^ ions or protons). While most secondary active transporters are composed only of integral membrane protein components, some transporter families have recruited a substrate-binding protein (SBP) that imparts directionality to transport and provides substrate affinities often in the nanomolar range, ideal for scavenging scarce nutrients (Rosa et al., 2018).

Tripartite ATP-independent periplasmic (TRAP) transporters are a large family of SBP-dependent secondary-active transporters that are found exclusively in prokaryotes to catalyze the uptake of a variety of substrates, primarily organic anions, across the cytoplasmic membrane (Mulligan et al., 2007, 2011). A defining feature of TRAP transporters is the obligate utilization of an SBP, which is either found in the periplasm or attached to the outer leaflet of the cytoplasmic membrane, where it binds and delivers the substrate to the membrane component. The membrane-embedded component of TRAP transporters consists of either two separate unequally sized integral membrane proteins, or a fusion of these two proteins into a single polypeptide with distinct domains (Kelly and Thomas, 2001).

TRAP transporters can be divided into two subtypes; the DctP-type, named after the first TRAP transporter characterized, which was specific to dicarboxylates (Forward et al., 1997), and the TRAP-associated extracytoplasmic immunogenic (TAXI)-type, named due to the immunogenic properties of the first one identified in *Bacillus abortus* (Mayfield et al., 1988; Kelly and Thomas, 2001). The membrane components of each of these TRAP subtypes share a low, but significant sequence identity of 10–15% (e.g., DctP-type HiSiaQM and TAXI-type PMI1055 are 13.25% identical, calculated using AlignMe [Stamm et al., 2013]), suggesting a shared lineage and likely the same overall fold. The subtype-specific SBPs on the other hand bear no relation to each other (Kelly and Thomas, 2001).

The vast majority of our understanding of TRAP transporter structure and function comes from the characterization of DctP-type TRAP transporters (Forward et al., 1997; Adnan et al., 2021; Deka et al., 2012; Herman et al., 2020; Kuhlmann et al., 2008; Meinert et al., 2017; Rabus et al., 1999; Rucktooa et al., 2007; Schäfer et al., 2021; Thomas et al., 2006; Vetting et al., 2015; Zhao and Binns, 2015; Bisson et al., 2022; Davies et al., 2023; Currie et al., 2024), most prominently, a sialic acid-specific member from *Haemophilus influenzae*, SiaPQM (HiSiaP is the SBP and HiSiaQM is the fused membrane component) (Mulligan et al., 2007, 2009, 2012; Currie et al., 2024; Peter et al., 2021, 2022; Severi et al., 2005; Fischer et al., 2015). Work from multiple labs on a variety of TRAP SBPs has revealed that, like SBPs from ATP-binding cassette (ABC) transporters, they utilize a venus flytrap type mechanism, in which the binding of ligand to the open *apo* state triggers closure of the two α/β globular domains around the substrate (Fischer et al., 2010). In addition, structural and phylogenetic analysis of DctP-type SBPs has revealed the presence of an extremely well-conserved arginine residue in the binding site that makes a critical salt bridge with the ligand, which in almost all cases is an organic acid (Fischer et al., 2010, 2015; Müller et al., 2006). Functional reconstitution of SiaPQM from *H. influenzae*, *Photobacterium profundum*, and *Vibrio cholerae* revealed that these transporters, and likely all DctP-type TRAPs use a Na^+^ gradient to power transport (Davies et al., 2023; Currie et al., 2024; Mulligan et al., 2009, 2012).

While there are dozens of DctP-type SBP structures available revealing a broad range of substrates (Rosa et al., 2018; Mulligan et al., 2011; Vetting et al., 2015), it was not until recently that experimentally derived structures of the membrane components were elucidated (Davies et al., 2023; Currie et al., 2024; Peter et al., 2022). Structural analysis of HiSiaQM from *H. influenzae* (PDB ID: 7QE5, 8THI, and 8THJ) and *P. profundum* (PDB ID: 8B01, 7QHA) has revealed important details regarding the interaction between the membrane component and the SBPs and the overall architecture of the membrane component. As previously predicted (Ovchinnikov et al., 2014), TRAP transporters share the same fold as members of the divalent anion Na^+^ symporter (DASS) family (Mancusso et al., 2012; Sauer et al., 2020, 2021; Mulligan et al., 2014), and both employ an elevator-like mechanism to transport substrate across the membrane (Sauer et al., 2020; Mulligan et al., 2016; Peter et al., 2024).

Compared to the DctP-type TRAPs, TAXI-TRAP transporters are relatively poorly studied despite being widespread in prokaryotes, and they differ in several ways. As with DctP-type TRAP transporters, TAXI-TRAPs are found in both bacteria and archaea, but TAXIs are the *only* type of TRAP transporter found in archaea, suggesting that TAXIs are the more ancient of the two subtypes (Kelly and Thomas, 2001). In addition, while the DctP-type transporter membrane components can be composed of two separate polypeptides or a fusion of the two, TAXI-TRAP membrane components are only ever a single fused polypeptide. Finally, functional reconstitution of an α-ketoglutarate specific TAXI from *Proteus mirabilis* reveals that, instead of being driven by Na^+^ electrochemical gradient as for the characterized DctP-type TRAPs, this TAXI is powered by a H^+^ gradient, which is a significant mechanistic schism in the family (Roden et al., 2023).

In contrast to DctP-type TRAPs, there are no published structures of TAXI membrane components, and there is only one published TAXI SBP structure, a glutamate or glutamine binding protein (TtGluBP) from *Thermus thermophilus* (PBD ID: 1US4 and 1US5) (Takahashi et al., 2004). However, the identity of the bound substrate was ambiguous and there was no further investigation into binding determinants. There is also an unpublished structure of a TAXI SBP from *Erlichia Chaffeensis* (PDB ID: 4DDD) in the PDB, which has a glycerol molecule and chloride ion bound in the vicinity of the binding site, but it is not clear whether these are substrates. A TAXI transporter from *Azoarcus* sp. CIB has been shown to transport orthophthalate (Sanz et al., 2020), and phenotypic analysis of a TAXI gene knockout in *Psychrobacter arcticus* suggests it is involved in the uptake of butyrate, glutamate, fumarate, and acetate (Bakermans et al., 2009). This limited number of examples hints that TAXIs can also transport a range of substrates akin to the DctP-type TRAPs. To provide more insight into the substrate range, structural arrangement, and key binding determinants of TAXI SBPs, we have obtained the structure of VC0430 from the human pathogen *V. cholerae*, determined the substrate specificity and affinity, and ascertained the effects of binding site mutation on ligand interactions. We have also investigated the interaction between the SBP and membrane components using modeling approaches revealing a hitherto unreported interfacial arrangement for any TRAP transporter.

## Materials and methods

### Molecular biology

Site-directed mutagenesis was performed using the Quikchange II kit (Agilent) or using KOD Hot-start DNA polymerase (Merck) followed by DpnI treatment. All plasmids and mutants were sequence verified prior to use.

### Expression of VC0430

To overexpress VC0430 for the periplasmic localization and ESI-MS analysis, the gene encoding VC0430 was expressed in BL21 (DE3) from a pET20b plasmid with the gene in-frame with a cleavable N-terminal PelB signal sequence and a C-terminal His_6_ tag. For all other analyses, the gene encoding VC0430 was overexpressed in BL21 (DE3) transformed with a pET-based plasmid with the gene in frame with an N-terminal FLAG/His_10_ tag (pETnHisVC0430) in place of the signal peptide (first 30 residues, MKEGKFMSLPKIIKMGAIAAAVIGSGVASA) (Love et al., 2010).

BL21(DE3) pETnHisVC0430 was grown in LB media supplemented with 50 µg/ml kanamycin and incubated at 37°C until an OD of 0.6–0.8 was reached, at which point expression was induced by the addition of 1 mM IPTG. The induced cells were incubated overnight at 37°C, harvested by centrifugation at 4,000 rcf for 20 min, resuspended in Purification Buffer (PB, 50 mM Tris, pH 8, 200 mM NaCl, 5% vol/vol glycerol), and lysed by three passes through a cell disruptor (Avestin). The lysate was clarified by centrifugation at 20,000 × *g* for 20 min at 4°C. BL21(DE3) pET20bVC0430 was treated similarly, except cells were grown at 25°C, were induced at an OD of 0.4, and were lysed using a French pressure cell.

### Purification of VC0430

The periplasmically located VC0430 that was used for ESI-MS analysis was purified by applying the clarified lysate to Ni-NTA resin (Qiagen), washing the resin with Wash Buffer (WB, PB containing 20 mM imidazole), and then eluting bound VC0430 with PB containing 300 mM imidazole. Eluted protein was further purified using size exclusion chromatography (SEC) prior to analysis. N-terminally FLAG/His-tagged VC0430, which was used for all other analyses, was denatured during the purification process to remove any pre-bound ligand. Clarified lysate was incubated with Ni-NTA resin, which was then washed with 30 column volumes (CV) of WB + 2 M guanidinium chloride (GdmCl) to denature the protein. Protein was refolded by subsequent washes with 4 CV WB + 1.5 M GdmCl, 4 CV WB + 1 M GdmCl, 4 CV WB + 0.5 M GdmCl, and finally 8 CV WB. Refolded protein was eluted with PB + 300 mM imidazole. The FLAG/His tag was cleaved for binding analysis and crystallography by incubation with TEV protease for 4 h at room temperature before reapplying the mixture to Ni-NTA resin and collecting the tag-free VC0430 in the flowthrough. For the protein used for crystallography, VC0430 was further purified using size exclusion chromatography with a Superdex 200 Increase 10/300 GL column (GE Healthcare).

### Oligomeric state analysis using SEC standards

For molecular weight measurements, Ni-NTA purified VC0430 was concentrated and applied to a Superdex 200 Increase column at a flowrate of 0.5 ml/min with SEC buffer (50 mM Tris, pH 7.5). The calibration curve was generated by analyzing SEC standards (Thermo Fisher Scientific) under than same conditions as VC0430. The partition coefficient (K_av_) for the standards and VC0430 using the equation: $K_{\text{av}}–\left(V_{e}–V_{o}\right)/V_{t}–V_{o}$, where V_e_ is the elution volume, V_o_ is the column void volume, and V_t_ is the total column volume.

### Differential scanning fluorimetry (DSF)

To perform DSF, 5 µM protein was mixed with the substrate (0, 1, or 15 mM) and 2.5× SYPRO Orange dye (Thermo Fisher Scientific), and made up to a total volume of 50 μl with DSF buffer (50 mM Tris pH 8, 20 mM NaCl). Using a QuantStudio 3 RT-PCR thermocycler (Invitrogen), the DSF samples were incubated at 5°C for 1 min, then the temperature increased to 95°C in 1°C increments, holding at each temperature for 10 s. The reporter dye setting used was set to SYBR. Melt curve data were exported to Microsoft Excel and GraphPad Prism for analysis and presentation.

### Tryptophan fluorescence spectroscopy

All tryptophan fluorescence assays were performed using a Cary Eclipse fluorimeter (Agilent). Fluorescence emission spectra were collected using 0.5 μM VC0430 in 50 mM Tris, pH 7.4, at 20°C, exciting at 295 nm wavelength with an emission range of 300–400 nm, slit widths set to 10 nm, and medium photomultiplier tube (PMT) voltage. Data were smoothed using a Savitzky-Golay smoothing factor of 15 for the initial emission scans. In most cases, ligand titrations were performed using 500 nM VC0430 in time-based acquisition mode with λex of 295 nm, λem of 330 nm, and excitation and emission slit widths of 5 and 10 nm, respectively. For higher affinity interactions (wildtype, E125A, Y200A, and M201A with L-glu), 10 nM VC0430 was used with slit widths set to 5 nm for the excitation slit and 20 nm for emission slit, and PMT voltage manually set to 900–925 V. Following baseline fluorescence collection for 30 s, the fluorescence change for each addition of ligand was measured for 20 s, which was averaged to calculate the ∆fluorescence for each addition. Binding curves were analyzed using GraphPad Prism and Kd values were obtained by fitting the curves to a one site-specific binding model:

$$Y=Bmax\times \frac{X}{\left(Kd+X\right)}.$$

### Mass spectrometry

For mass spectrometry, SEC-purified VC0430 was dialyzed against 50 mM sodium phosphate, pH 8, dialyzed against water, and then concentrated prior to analysis. Electrospray mass spectrometry was performed using the API Qstar mass spectrometer using an ionspray source. To determine the mass of the unliganded protein, 1 μM VC0430 was made up of acetonitrile and 0.1% formic acid. To determine the mass of the protein with bound ligand, 10 μM VC0430 was made up of 25 mM ammonium acetate, pH 4.5, and 3% (vol/vol) methanol. Raw m/z data were deconvoluted using the Bayesian Protein Reconstruction routine.

### Protein crystallography

VC0430 (15 mg/ml final concentration) was incubated with 0.5 mM L-glutamate before being mixed in a 1:1 ratio with 2.4 M sodium malonate at pH 7.0 and (JCSG+ Condition F9 with no changes made to the solution), over a well of the same buffer in a hanging drop vapor diffusion plate. For x-ray diffraction, the crystals were then picked using a 0.3–0.4 µm nylon loop. The crystals were immediately flash frozen in liquid nitrogen. X-ray diffraction experiments were conducted at Diamond Light Source using the macromolecular beam i04 with a wavelength of 0.9537 Å. Data were collected over a 360° rotation of the omega axis, collecting images at 0.1°. The full statistics for the crystals can be found in Table S3. In brief, the crystals diffracted to ∼1.7 Å with a CC1/2 of 1.0 over the data range. The crystals had a space group of R32 and the data collected were used to solve the structure using the Diamond Light Source automated pipeline. The data were indexed, integrated, and scaled by the xia2 3dii pipeline (Winter, 2010). The processed data was then used for a molecular replacement model obtained through AlphaFold2 (Jumper et al., 2021) and autobuilding using Dimple (Wojdyr et al., 2013). The initial structure consisted of 300 built-in residues; additional building, additional residue, and water placement were performed using Coot; and Phenix was used for refinement and automated water placement (Emsley et al., 2010; Liebschner et al., 2019). All images were generated using UCSF ChimeraX (Pettersen et al., 2021).

### Bioinformatics

A total of 59 TAXI amino acid sequences from a wide range of species were obtained from Uniprot and aligned using MAFFT from EMBL-EBI (Madeira et al., 2022). The alignment was visualized in Geneious Prime 2022.2.1 (https://www.geneious.com) and consensus residues were highlighted. To construct the maximum-likelihood phylogenetic tree, alignment gaps at the N- and C-terminus were trimmed. A phylogenetic tree was created using IQ-TREE with 1,000 replicates of ultra-fast bootstrapping approximation (Nguyen et al., 2015). The unrooted consensus tree was then visualized using the Interactive Tree of Life (iTol) tool (Letunic and Bork, 2021).

### Online supplemental material

**Figure S1.**
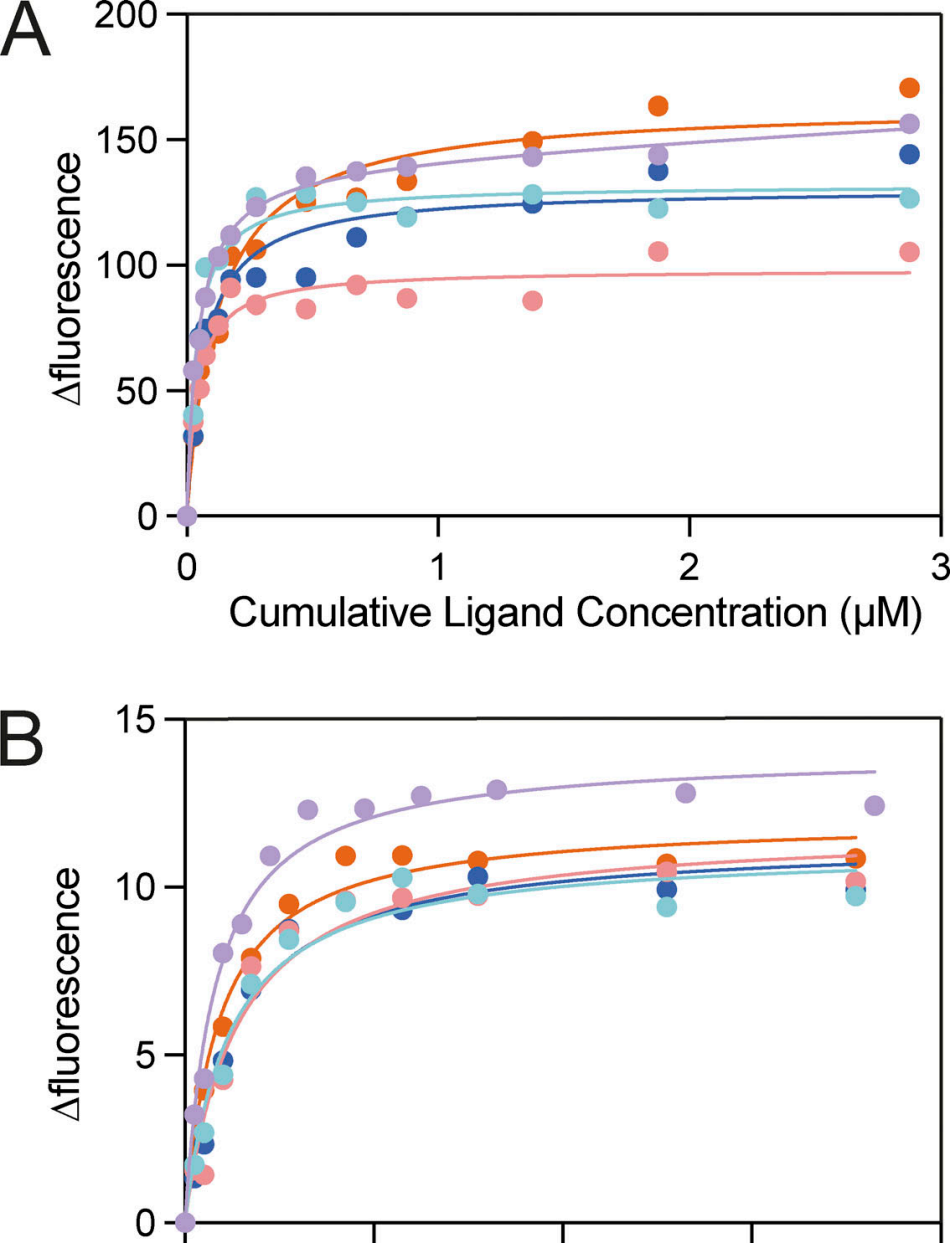
**VC0430 binding curves for L-glu and L-gln. (A and B)** Replicate data for VC0430 wildtype binding to (A) L-glutamate and (B) L-glutamine. Each color represents a different technical replicate.

**Figure S2.**
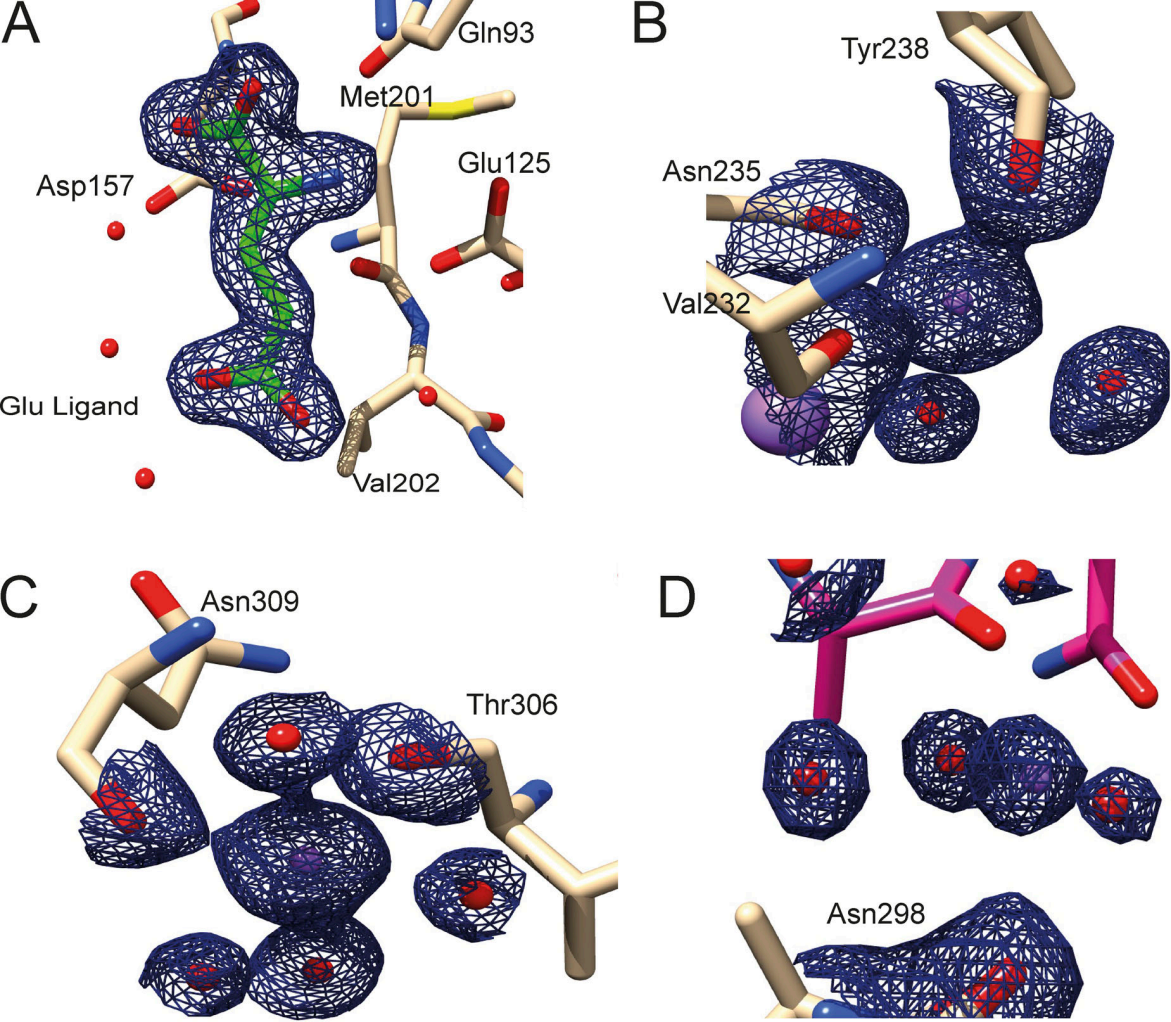
**Electron density of bound ligand in VcGluP crystal structure. (A)** Electron density (black mesh) of the bound L-glutamate. **(B–D)** Electron density (black mesh) for three Na^+^ ions (purple spheres). Water molecules are shown as red spheres. A–C density contoured at 1.65 σ and D is contoured at 1.4 σ.

**Figure S3.**
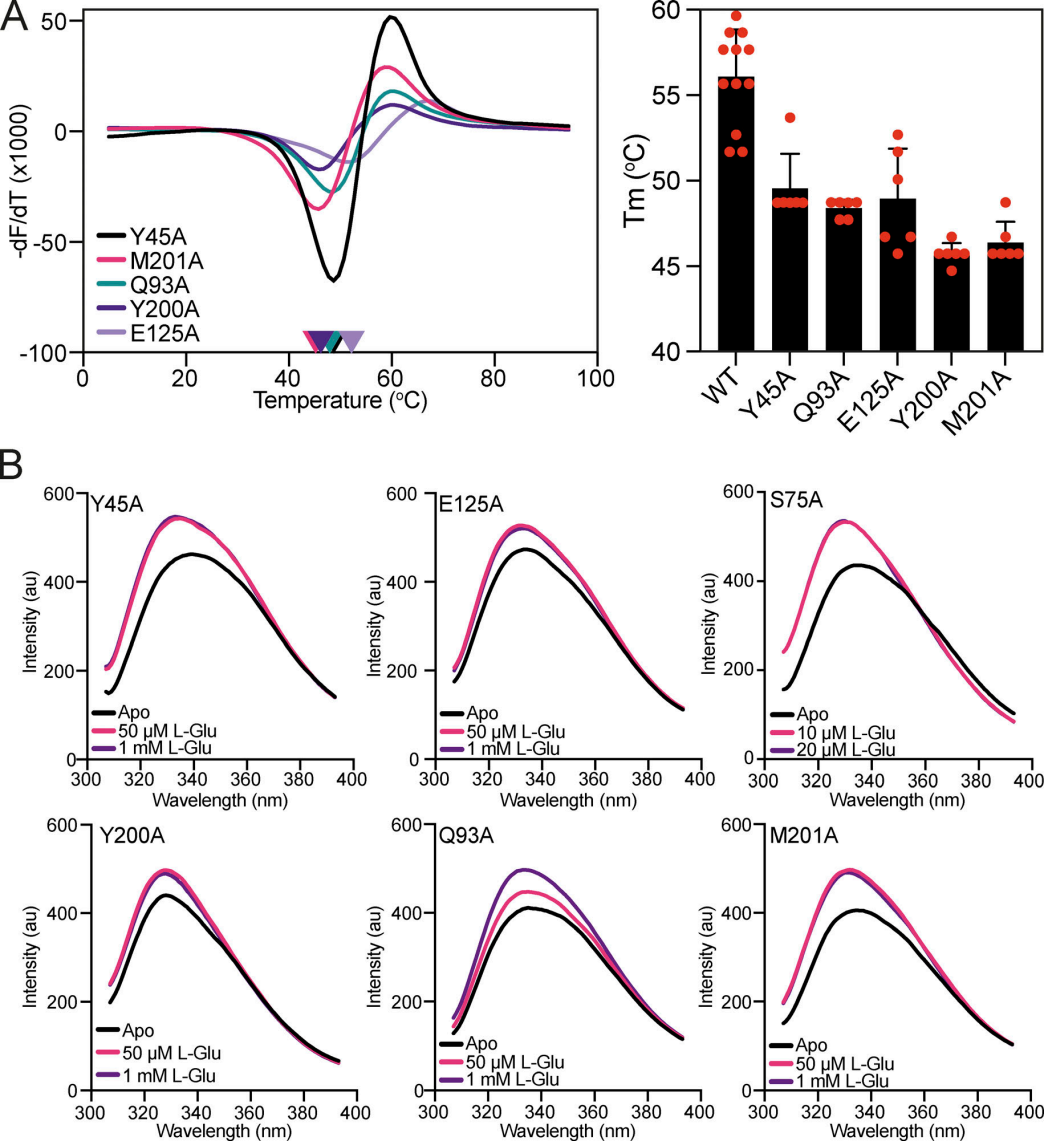
**Thermostability and tryptophan fluorescence analysis of VcGluP binding site mutants. (A)** Melting temperature of each VcGluP binding site mutant. Left panel: Representative DSF data for each VcGluP binding site mutant in the absence of substrate. Right panel: The Tm of each binding site mutant derived from the DSF assay. Error bars represent standard deviation and individual datapoints are shown in red. **(B)** Emission scans of each mutant with an excitation wavelength of 295 nm in the presence and absence of L-glutamate.

**Figure S4.**
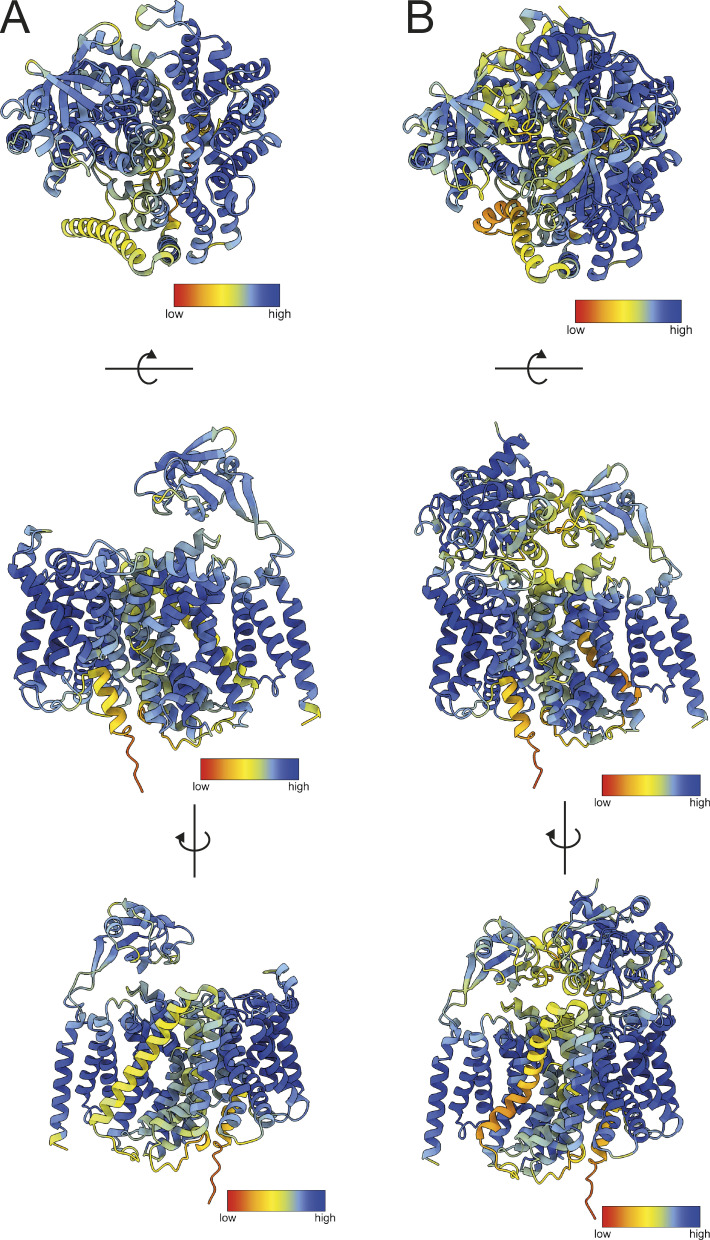
**VcGluPQM and VcGluQM model prediction confidence. (A and B)** AlphaFold2 model of (A) VcGluQM and (B) Colabfold model of VcGluPQM complex colored by pLDDT score. Models oriented for top-down view (upper panels), side view (middle panel), and alternative side view rotated 180° (lower panel). pLDDT score key is indicated in each panel.

**Figure S5.**
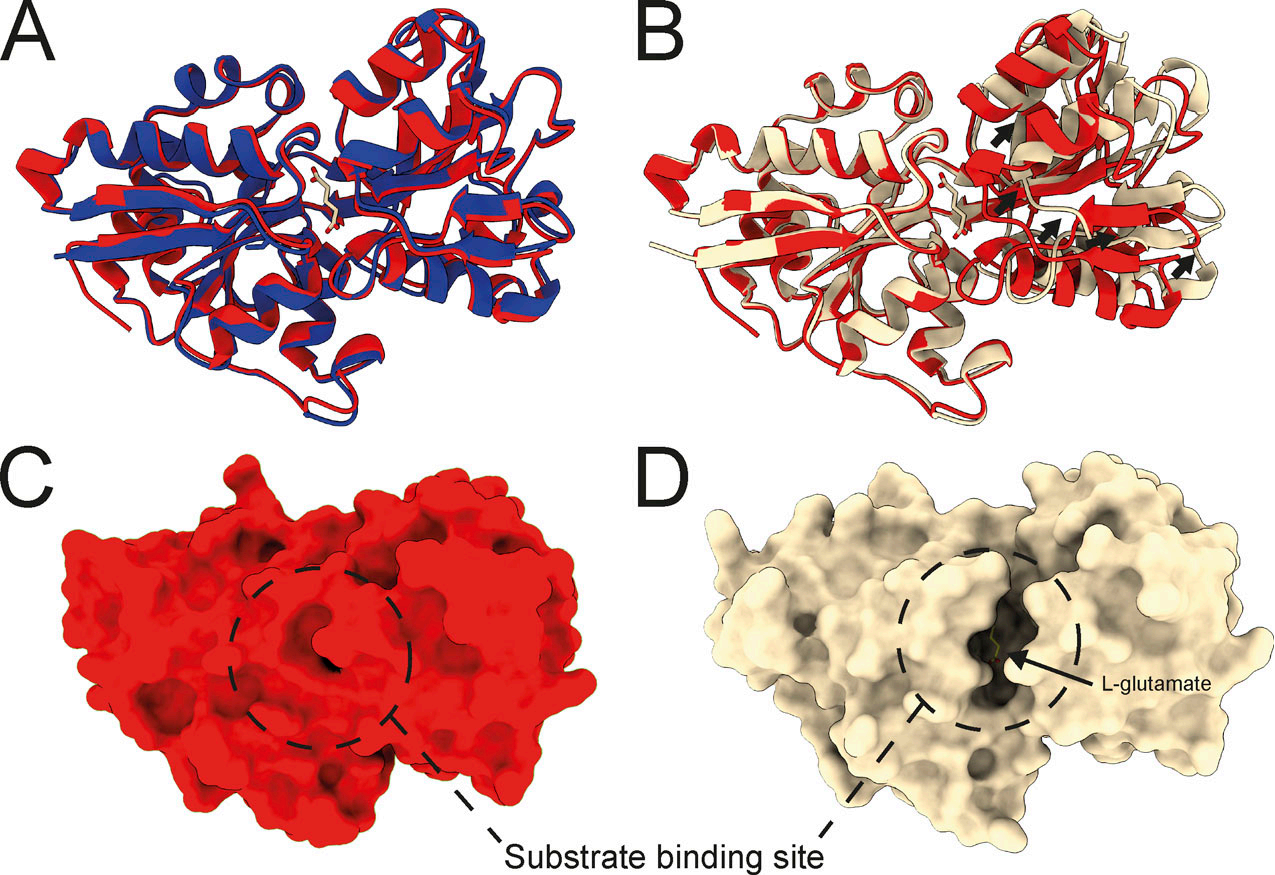
**Structural alignments of VcGluP structural models. (A)** Superimposition of the cartoon representations of VcGluP crystal structure (red) or Alphafold2 model (blue). **(B)** Superimposition of the cartoon representations of VcGluP crystal structure (red) or model derived from VcGluPQM Colabfold model (wheat). **(C)** Surface representation of the VcGluP crystal structure showing no access to the binding site. **(D)** Surface representation of the VcGluP derived from the VcGluPQM Colabfold model showing a solvent-accessible binding site.

**Figure S6.**
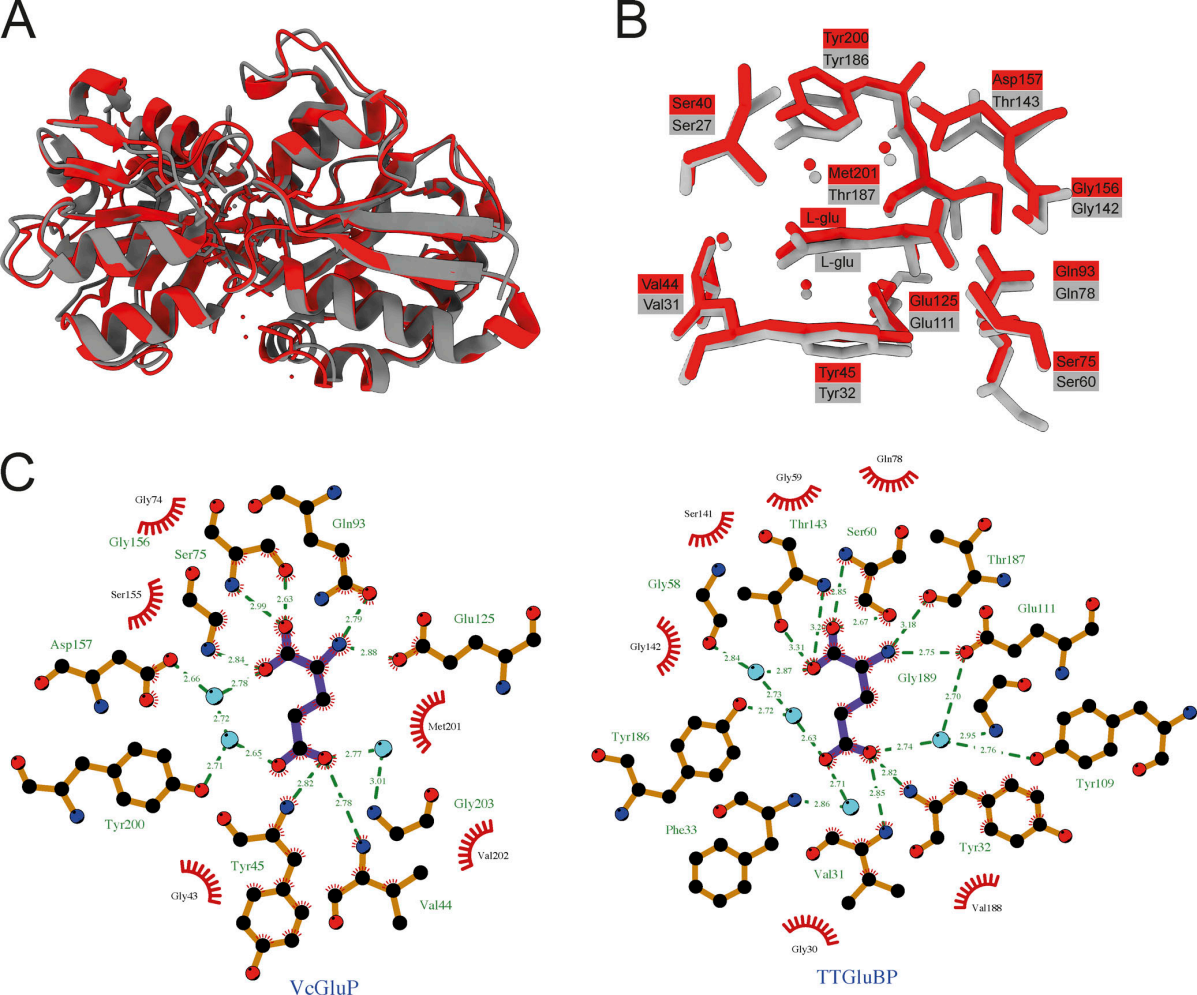
**Structural comparison of VcGluP and TtGluBP. (A)** Cartoon representation of superimposition of VcGluP (red) and TtGluBP (grey) with an RMSD of 2.1 Å. **(B)** Binding site arrangement of VcGluP (red) and TtGluBP (grey). Residues and bound ligand are labeled and color coded. **(C)** LigPlot+ representation of the binding sites of VcGluP (left) and TtGluBP (right).

**Figure S7.**
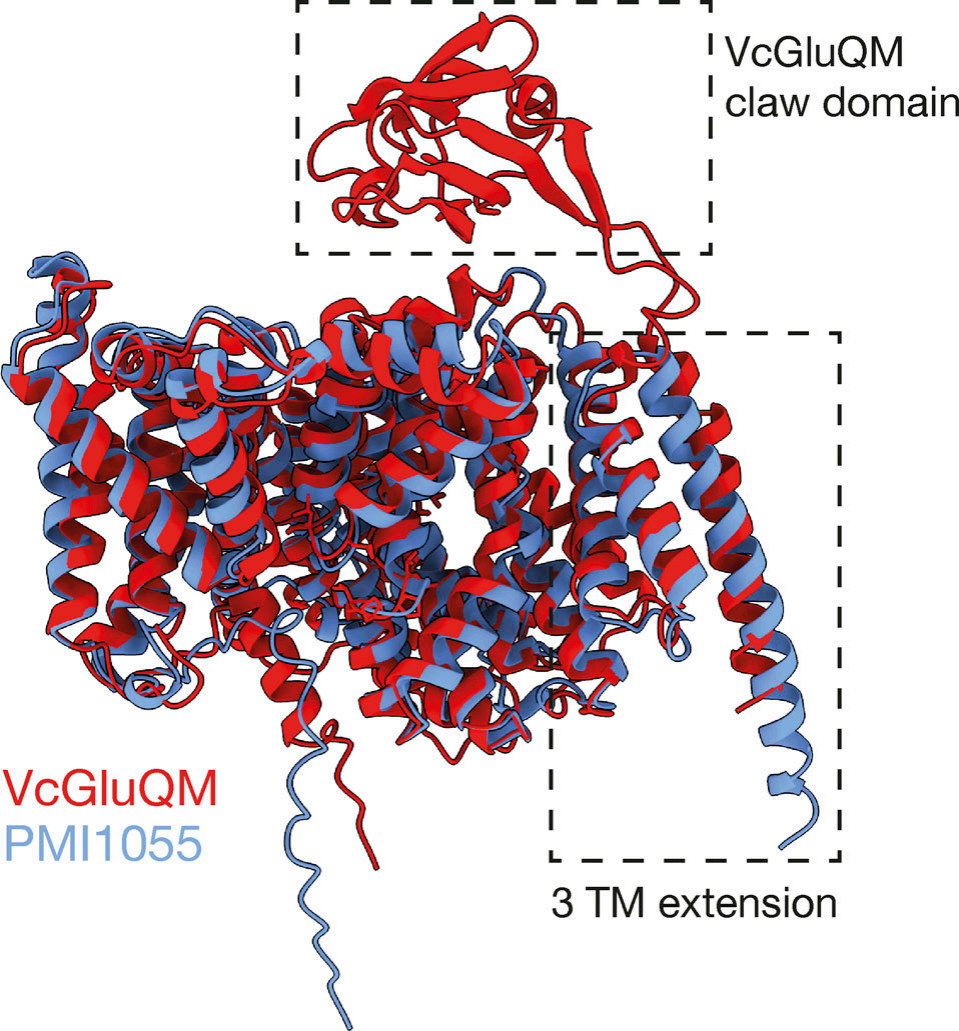
**Structural alignment of the Alphafold2 models of VcGluQM (red) and PMI1055 (blue).** The three transmembrane helix (3 TM) extension present in both proteins is highlighted. The periplasmically located claw domain of VcGluQM is also highlighted.

Fig. S1 shows the replicates for the L-glu and L-gln binding curves for VC0430 wildtype summarized in Fig. 4. Fig. S2 is the electron density of bound ligand in the VcGluP structure. Fig. S3 shows the DSF traces and fluorescence emission scans for VcGluP mutants. Fig. S4 shows the VcGluPQM models colored by pLDDT score. Fig. S5 shows a structural alignment of VcGluP models. Fig. S6 shows a comparison of VcGluP with TtGluBP. Fig. S7 shows the structural alignment of VcGluQM and PMI1055. Data S1 is a multiple sequence alignment of a variety of TAXI binding proteins. Table S1 lists *E. coli* metabolites between 144 and 148 Da. Table S2 shows full datasets from DSF binding analysis presented in Fig. 3 and Fig. 5. Table S3 shows data collection and refinement statistics.

## Results

### Bioinformatic analysis of VC0430

**Figure 1.**
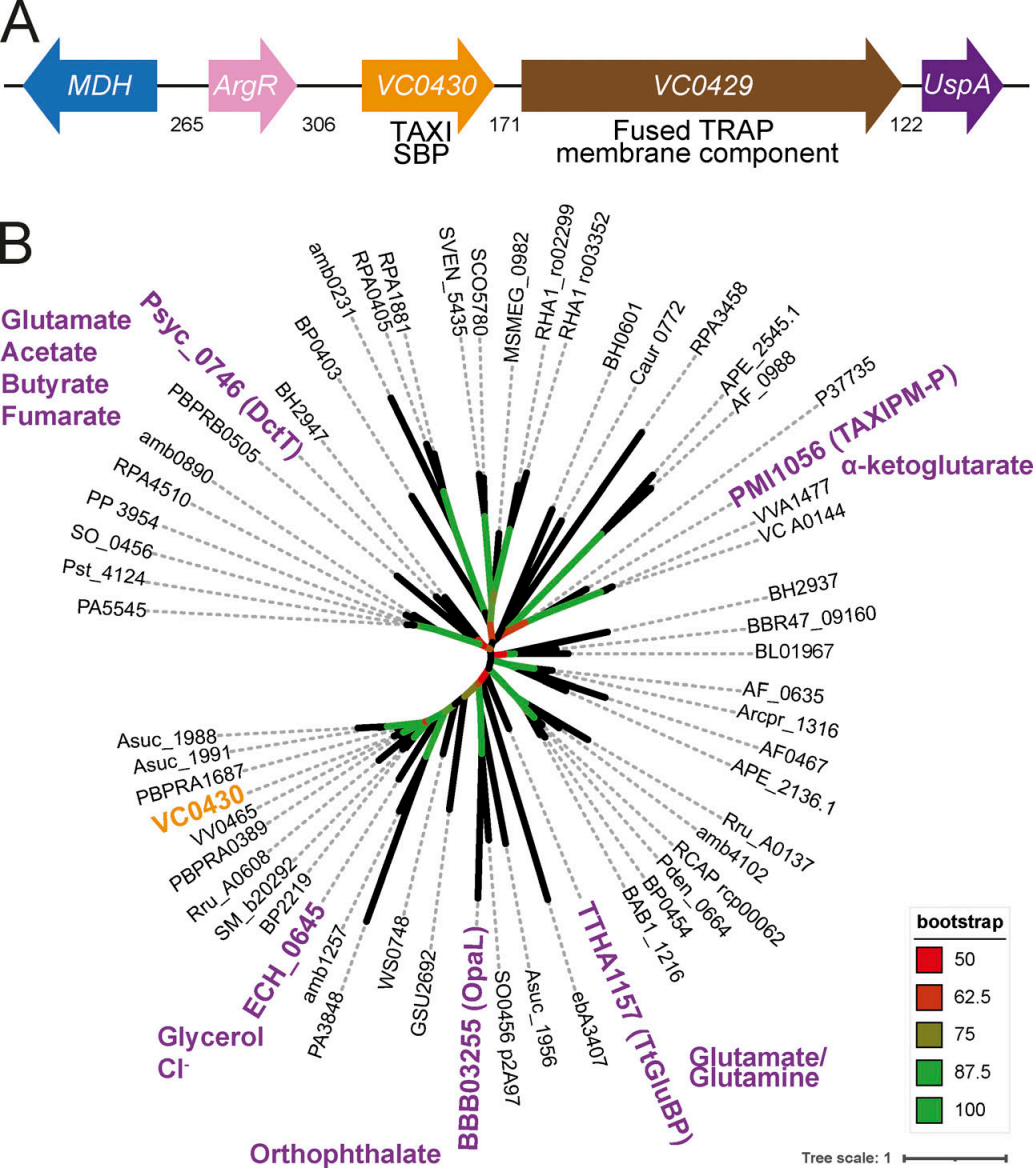
**Genome context and phylogenetic analysis of VC0430. (A)** Genome context of *VC0430*. Numbers indicate the number of intergenic basepairs. **(B)** Phylogenetic tree of 59 homologs of VC0430 (locus tag for each protein is displayed). The TAXI SBPs that have been characterized to any degree have been highlighted in purple and the known or predicted substrate(s) are indicated. VC0430 in orange is the subject of this study. The accompanying sequence alignment is presented in Data S1.

To characterize VC0430, we first sought to identify its ligand. As a starting point, we analyzed the genome context of *VC0430*, to see if characterized co-regulated genes could provide clues to the identity of the ligand; a strategy used with much success previously (Herman et al., 2020; Thomas et al., 2006). As expected, the gene encoding VC0430 is adjacent to the gene encoding its cognate membrane component, VC0429 (Fig. 1 A). The TRAP transporter genes are downstream of two other genes, *argR* and *mdh* which encode an arginine repressor and malate dehydrogenase, respectively (Fig. 1 A) (Lim et al., 1987). Arginine and malate both contain carboxyl groups, which is a common feature of known TRAP ligands, raising the possibility that they are substrates of the transporter, but the lack of support for coregulation (based on annotation from MicrobesOnline [Dehal et al., 2010]) between these genes and the TRAP genes diminishes the strength of this prediction. The gene downstream of the TRAP genes encodes a universal stress protein (UspA); the association between TRAP transporters and UspA has been noted previously (Mulligan et al., 2011; Schweikhard et al., 2010), and while they appear to negatively regulate TRAP transporter function, their physiological role and mechanism are not known (Schweikhard et al., 2010).

An alternative approach to predict the substrate of orphan transporters is to see if any closely related SBPs are characterized because they may bind the same or related ligands. To perform this analysis, we collected and aligned the amino acid sequences of 59 TAXI SBPs from a variety of organisms, making sure to include any characterized TAXI SBPs (Fig. 1 B). The phylogenetic tree produced by this alignment revealed several clades. However, when the positions of the TAXIs with known or predicted substrates were mapped onto the tree, none were sufficiently closely related to VC0430 to permit any predictions (Fig. 1 B). In the absence of strong evidence for the identity of VC0430’s ligand from bioinformatic approaches, we sought to identify the ligand experimentally. As SBPs generally have a high affinity for their cognate ligand, SBPs can retain the bound ligand during the purification process if that ligand is present in the expression strain. Therefore, we decided to identify VC0430’s ligand by expressing and purifying the protein and screening for the cognate ligand using mass spectrometry, an approach used previously to de-orphanise SBPs (Herman et al., 2020; Thomas et al., 2006; Vetting et al., 2015).

### VC0430 is monomeric and interacts with both glutamate and glutamine

**Figure 2.**
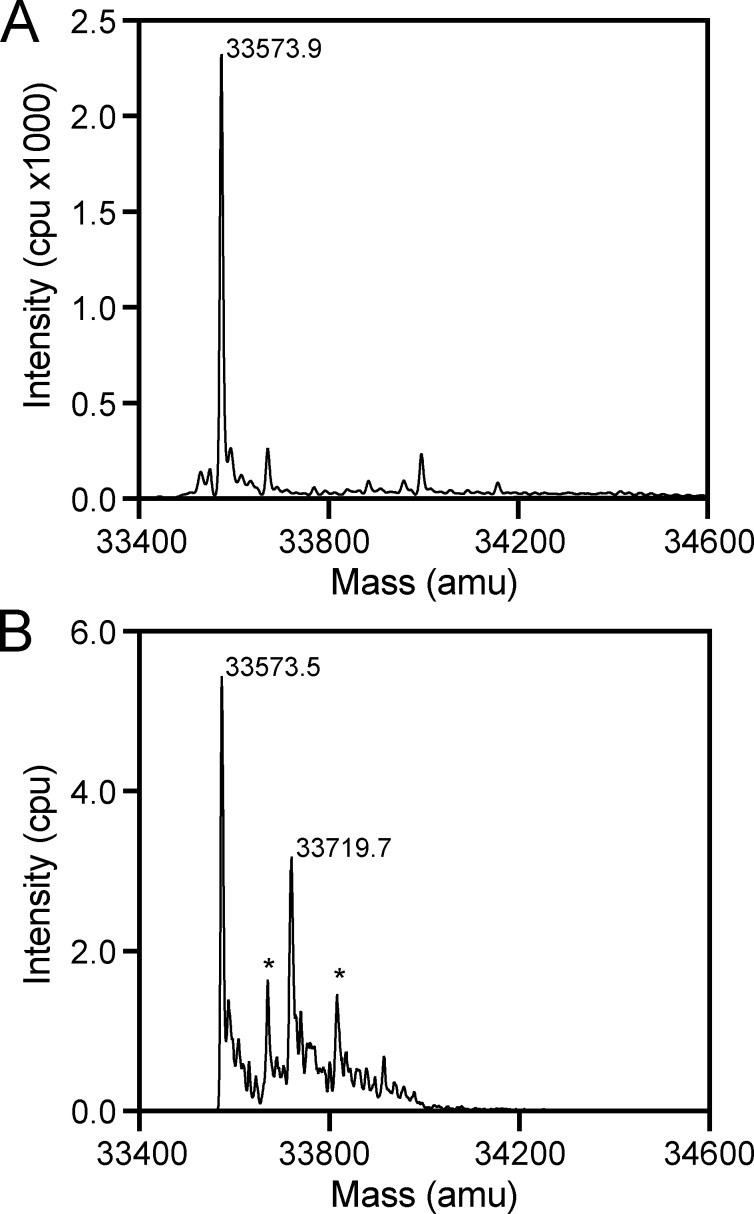
**Identification of ligand mass using mass spectrometry. (A)** Denaturing MS analysis of VC0430 reveals a single peak corresponding to a mass of 33,573.9 Da. **(B)** Native MS analysis of VC0430 reveals major peaks corresponding to masses of 33,573.9 and 33,719.7 Da, which represent the *apo* and ligand bound VC0430, respectively. Peaks labeled with an * correspond with phosphate adducts of the major peaks.

To identify any ligands bound to VC0430 using this mass spectrometry approach, we overproduced VC0430 in *E. coli* BL21 (DE3) with an N-terminal cleavable signal peptide and a C-terminal His_6_-tag, which would translocate the protein to the periplasm, and facilitate purification, respectively. We purified VC0430 in one step with immobilized metal affinity chromatography (IMAC) and subjected it to mass spectrometry analysis under denaturing conditions, revealing a mass of 33,573.9 Da, which correlates well with the predicted mass of the mature protein with the C-terminal affinity tag (33,578 Da, Fig. 2 A). Under more native conditions, we observed two major peaks in the mass spectrum, the first with a mass of 33,573.5 Da which is essentially identical to the mass of the denatured protein suggesting it corresponds to the *apo* protein (Fig. 2 B). The second major peak had a mass of 33,719.7 Da, which we reasoned corresponded to ligand-bound VC0430 (Fig. 2 B). Calculating the difference between the two major MS peaks under native conditions revealed a mass difference of 146.2 (±2 Da), providing us with an approximate mass of the bound ligand; an important clue in its identification. Two smaller peaks were present that were spaced 98 Da from each of the main peaks suggesting that they are phosphate adducts (Fig. 2 B).

Armed with the approximate mass of the ligand (144–148 Da), we narrowed the list of potential compounds further by restricting our search to metabolites present in *E. coli* using the EcoCyc (Keseler et al., 2021). This search resulted in a list of 51 different compounds (Table S1).

**Figure 3.**
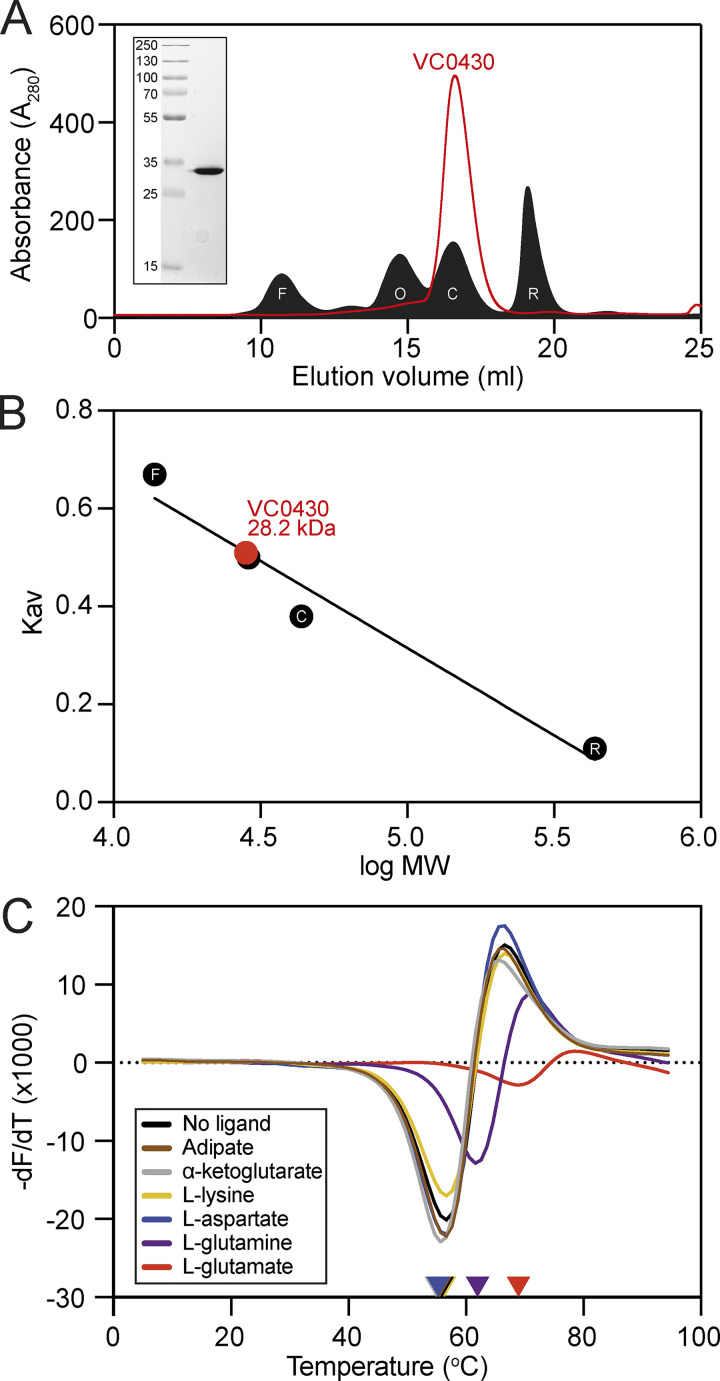
**Purification, analytical size exclusion chromatography, and ligand binding of VC0430. (A)** SEC elution profile of VC0430 (red line) compared to the profiles of four standards (filled black trace); ferritin (F, 10.67 ml elution volume), ovalbumin (O, 14.71 ml), carbonic anhydrase (C, 16.52 ml), and ribonuclease A (R, 19.08 ml). **(B)** Molecular weight calibration curve showing Kav as a function of the log MW of the four standards from A (black circles, labels are the same as in A). Comparison of the VC0430’s Kav reveals an MW of 28.2 kDa. **(C)** Derivatives of the unfolding curves (dF/dT) for VC0430 in the absence of ligand and the presence of 1 mM adipate, a-ketoglutarate, L-aspartate, L-glutamate, L-glutamine, and L-lysine. Colored arrow on the X-axis indicates the apparent protein Tm under those conditions. Information regarding replicates can be found in Table S2. Source data are available for this figure: SourceData F3.

To increase our yield of VC0430 for structural and biochemical studies, we changed the expression construct and replaced the N-terminal signal peptide with a TEV-cleavable decahistidine tag, thus leading to cytoplasmic expression. VC0430 was overexpressed using this new construct, denatured and renatured to remove as much prebound ligand as possible, and purified using IMAC and size exclusion chromatography (SEC), which revealed a single band in SDS-PAGE and a sharp, symmetrical peak in the SEC, indicative of well folded and stable protein (Fig. 3 A). While the vast majority of structurally characterized TRAP SBPs are monomeric, there are some notable exceptions that form stable dimers (Gonin et al., 2007). Therefore, to assess the oligomeric state of VC0430, we compared the elution volume of VC0430 from the SEC column to standards with known molecular weights (Fig. 3 A). Analysis of the calibration curve revealed that VC0430 has a mass of 28.2 kDa (Fig. 3 B), which is slightly smaller than the molecular weight predicted from the amino acid sequence (35.9 kDa), but it demonstrates that VC0430 exists as a monomer in solution.

To screen our list of compounds to see which could bind to VC0430, we used differential scanning fluorimetry (DSF). In this assay, the protein is thermally denatured in the presence of SYPRO Orange dye, the fluorescence of which increases as it binds to the hydrophobic core of the protein that is revealed during denaturation. DSF provides a read-out of the melting temperature (Tm) of the protein, which often increases upon ligand binding, thus providing a convenient way of detecting interactions.

Using DSF, we first screened the most conveniently available compounds from our EcoCyc-derived list of potential ligands; adipate, α-ketoglutarate, L-aspartate, L-glutamate, L-glutamine and L-lysine. Analysis of the first derivative of the fluorescence as a function of the temperature (dF/dT) for VC0430 alone revealed a Tm of 57.6°C (Fig. 3 C). In the presence of 1 mM L-aspartate, L-lysine, α -ketoglutarate, or adipate, we observed no change in VC0430’s Tm suggesting that these compounds do not bind (Fig. 3 C). However, upon addition of 1 mM L-glutamate and L-glutamine, we observed a substantial rightward-shift of the melt peak, with a Tm increase of 11.3°C and 5.6°C, respectively, indicating that both L-glutamate and L-glutamine bind to VC0430 (Fig. 3 C).

### Determining the binding affinity of VC0430

**Figure 4.**
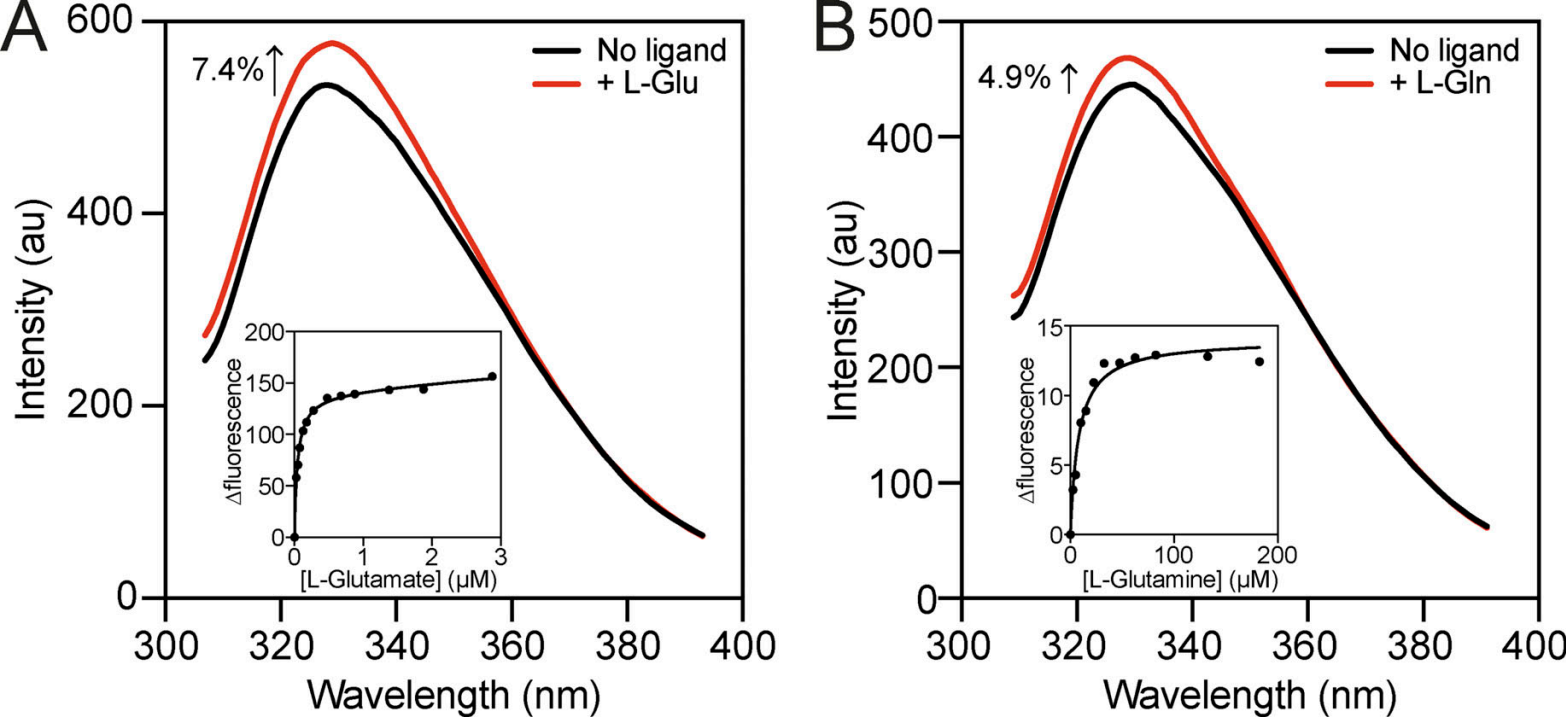
**Ligand binding affinity determination using intrinsic tryptophan fluorescence. (A and B)** Fluorescence emission scans of VcGluP in the absence of ligand (black lines) and in the presence of A 5 μM L-glutamate and B 100 μM L-glutamine (red lines). Inset: Representative L-glutamate (A) and L-glutamine (B) binding curves for VcGluP showing hyperbolic dose response. Single data sets for the titration (inset) are shown; replicate binding curve data are presented in Fig. S1.

To provide support for L-glutamate and L-glutamine being ligands of VC0430, we determined VC0430’s binding affinity for each compound using intrinsic tryptophan fluorescence. When excited at 295 nm, VC0430 exhibited a characteristic tryptophan emission spectrum between 300 and 400 nm with an emission maximum of ∼330 nm (Fig. 4, A and B, black line). Upon the addition of 5 μM L-glutamate, we observed a substantial 7.4% enhancement of the fluorescence at 330 nm, and a smaller 4.9% enhancement upon the addition of 100 μM L-glutamine, indicative of binding (Fig. 4, A and B, red line). To determine the binding affinities for L-glutamate and L-glutamine, we monitored the dose-dependent enhancement of fluorescence in the presence of increasing concentrations of each ligand (Fig. 4, A and B, inset and Fig. S1). Fitting the curves to a one site binding model revealed dissociation constants (Kd) of 0.065 ± 0.031 µM and 11.00 ± 2.19 µM for L-glutamate and L-glutamine, respectively. These Kd values are consistent with the affinities measured for other DctP-type TRAP SBPs and the other TAXI SBP that has been characterized (Mulligan et al., 2011; Roden et al., 2023). As these data suggest that L-glutamate is the preferred ligand for VC0430, we propose that VC0430 should be designated VcGluP (with the cognate membrane component Vc0429 designated VcGluQM), to remain consistent with the naming of DctP-type TRAP transporters.

### VcGluP stereoselectively binds its ligands

**Figure 5.**
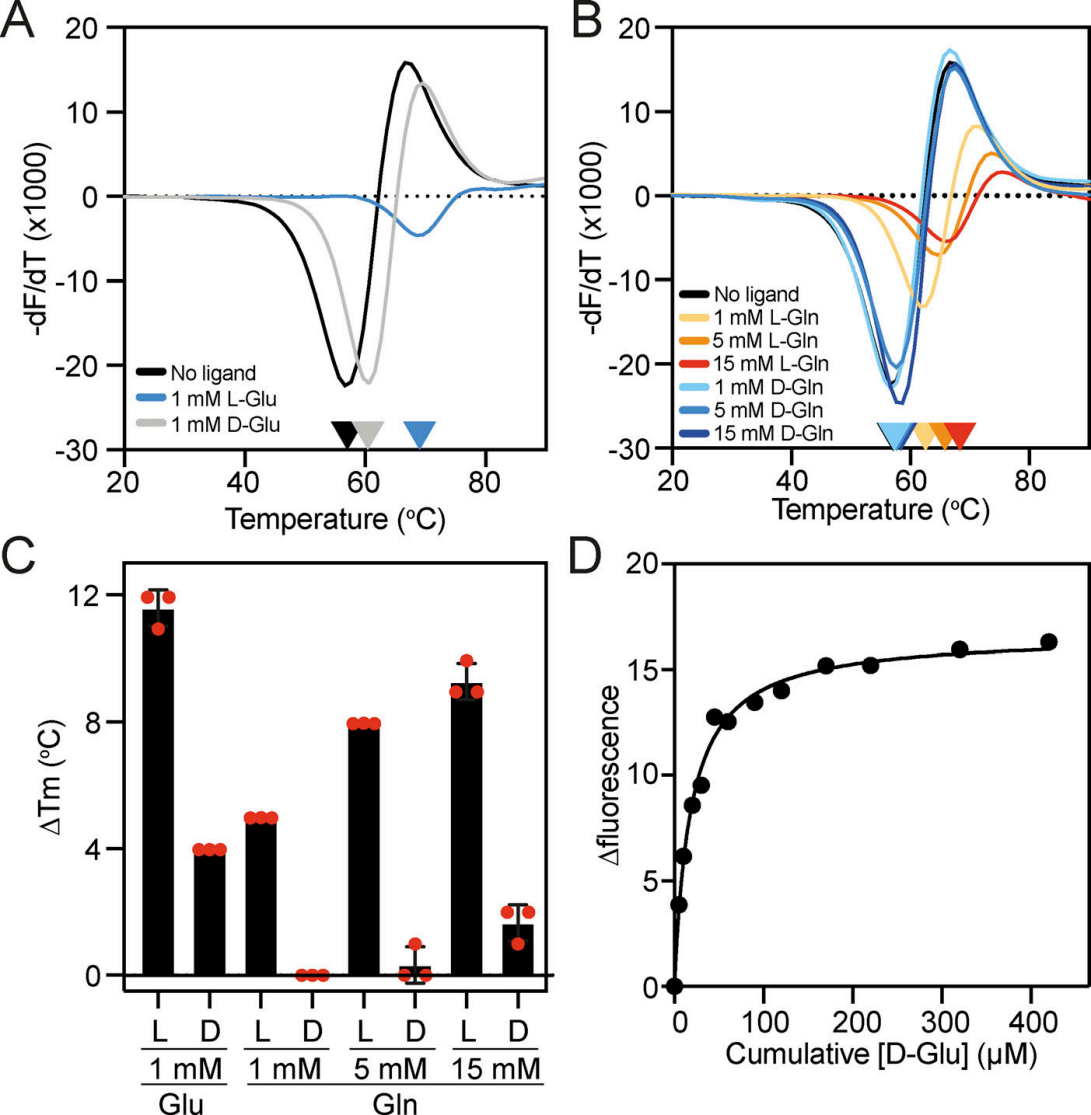
**Assessing the stereoselectivity of VcGluP. (A and B)** Derivatives of the unfolding curves (dF/dT) for VC0430 in the absence of ligand and the presence of (A) 1 mM L- and D-glutamate, and (B) 1, 5, and 15 mM L- and D-glutamine. **(C)** Thermostabilization of VcGluP in the presence of L- and D-glutamate and glutamine. Error bars represent standard deviation and individual data points are shown as red circles. **(D)** Representative binding curve for D-glutamate binding to VcGluP. Information regarding replicates of the unfolding curve in A and B can be found in Table S2.

Having identified that VcGluP binds both L-glutamate and L-glutamine, we next assessed VcGluP’s stereoselectivity for these ligands by evaluating its ability to bind D-glutamate and D-glutamine. DSF analysis revealed that, while L-glutamate induces a sizeable 11.6°C increase in thermostability of VcGluP, 1 mM D-glutamate induces lower but above background stabilization of 4.0°C (Fig. 5, A and C). D-glutamine has minimal effects on VcGluP stabilization, only inducing ∼1.7°C stabilization upon the addition of 15 mM (Fig. 5, B and C). To provide further insight into the stereoselectivity, we determined the binding affinity for the D-enantiomers using tryptophan fluorescence revealing a Kd of 24.5 ± 10.6 µM for D-glutamate (Fig 5 D). However, a Kd for D-glutamine could not be determined due to a lack of fluorescence enhancement. These data demonstrate that L-glutamate is VcGluP’s preferred ligand, but it is still able to bind the D enantiomer with an affinity in the range seen for other SBPs.

### L-pyroglutamate is a low-affinity ligand of VcGluP

**Figure 6.**
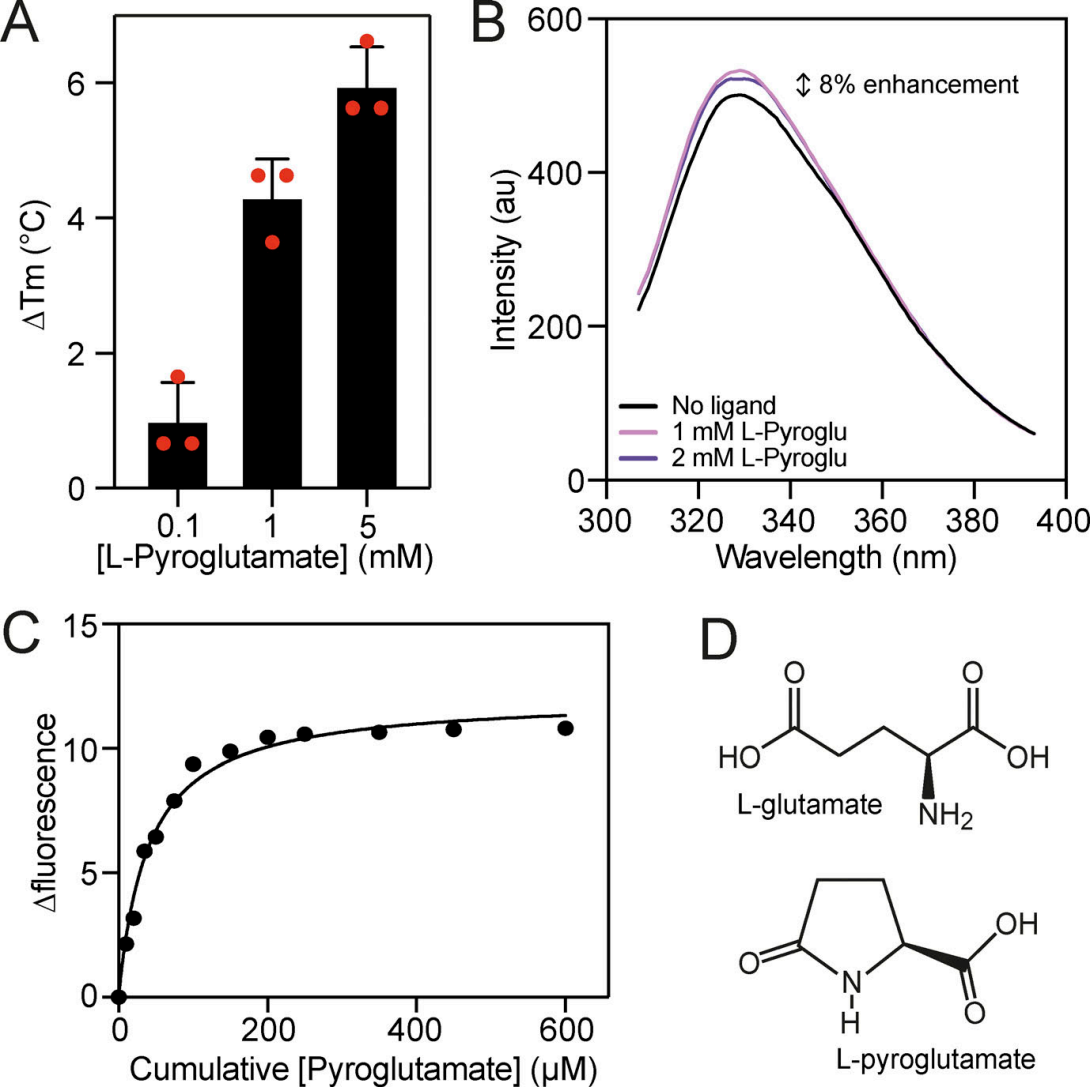
**VcGluP can also bind L-pyroglutamate. (A)** Thermostabilization of VcGluP in the presence of 0.1, 1, and 5 mM L-pyroglutamate. Error bars represent standard deviation and individual data points are shown as red circles. **(B)** Fluorescence emission scans of VcGluP in the presence and absence of L-pyroglutamate. **(C)** Representative binding curve for L-pyroglutamate binding to VcGluP. **(D)** Chemical structures of L-glutamate and L-pyroglutamate.

Due to the high affinity for L-glutamate exhibited by VcGluP and evidence of four other transporter-related SBPs binding the similar molecule L-pyroglutamate (Rucktooa et al., 2007; Vetting et al., 2015), we tested whether VcGluP could also bind L-pyroglutamate. DSF screening revealed that the addition of L-pyroglutamate enhanced VcGluP’s stability in a dose-dependent manner (Fig. 6 A), suggesting an interaction. In support of this, we observed an 8% enhancement in VcGluP’s intrinsic tryptophan fluorescence upon the addition of 1 mM L-pyroglutamate, which did not increase upon further additions, suggesting saturable binding (Fig. 6 B). We investigated the affinity of L-pyroglutamate binding using tryptophan fluorescence revealing a Kd of 50.0 ± 16.0 µM (Fig. 6 C). Therefore, while the affinity for L-pyroglutamate is >750× lower than the affinity for L-glutamate, VcGluP can clearly bind L-pyroglutamate (Fig. 6 D).

### Structural characterization of L-glu bound VcGluP reveals binding site residues

**Figure 7.**
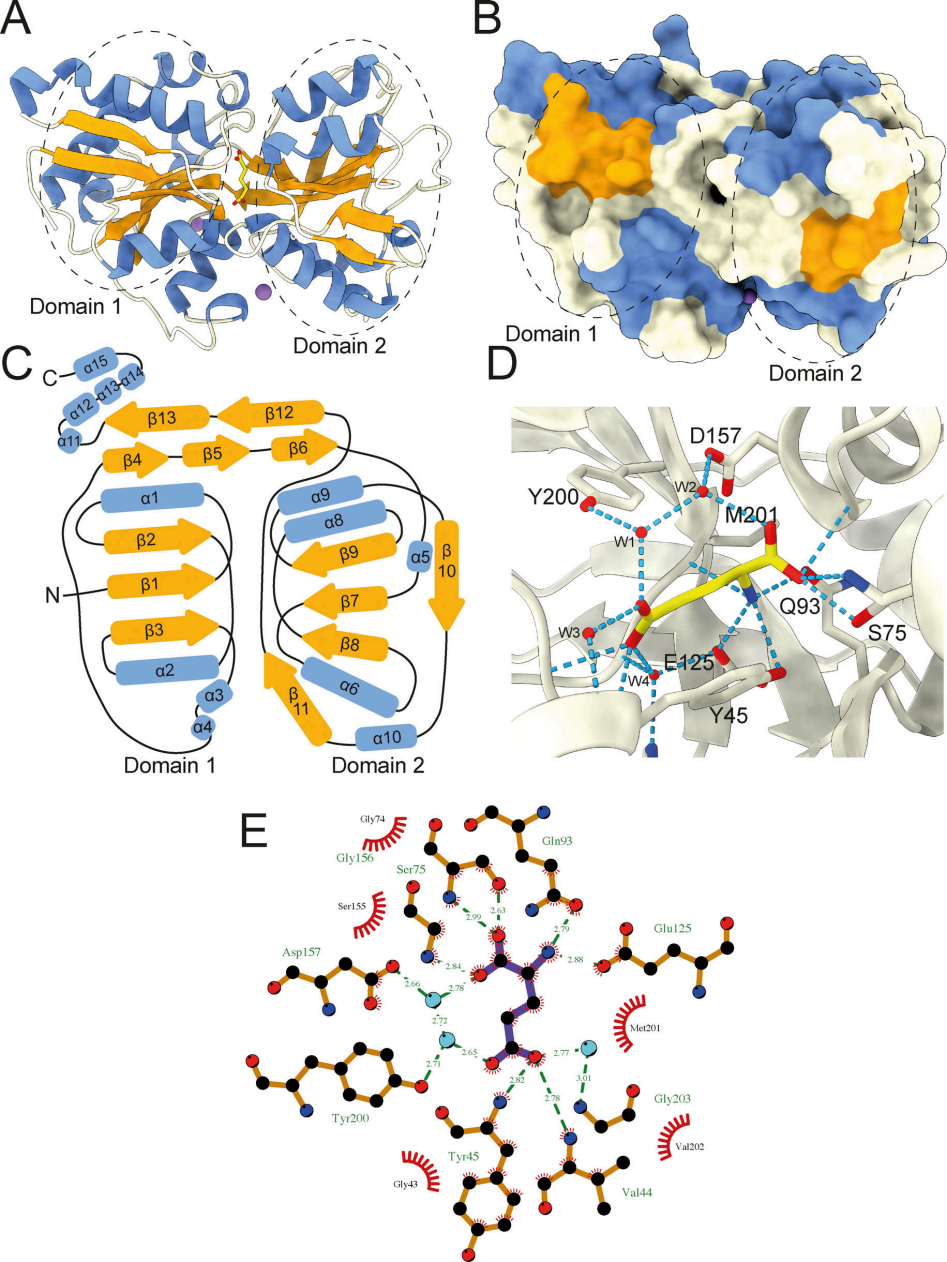
**Structural characterization of L-glutamate bound VcGluP. (A)** Cartoon representation of VcGluP colored according to secondary structure, α-helices in blue, β-sheets in orange, loops in white. The bound L-glutamate is shown in yellow in the central cavity and the Na^+^ ions are purple spheres. Domains 1 and 2 are demarcated by dashed lines. **(B)** Surface representation of VcGluP colored and in the same pose as in A. The lack of solvent accessibility to the binding site indicates that VcGluP is in a closed state. **(C)** Topology map of VcGluP, colored as in A. **(D)** Ligand binding site of VcGluP showing the interactions with the bounds L-glutamate. **(E)** LigPlot+ representation of the ligand binding site (ligand is purple sticks and water molecules are light blue circles).

To investigate the selectivity determinants of VcGluP, we solved the structure of VcGluP in the presence of excess (0.5 mM) L-glutamate to 1.7 Å, using molecular replacement (Fig. 7 A). In parallel, we attempted to elucidate the structure of *apo* VcGluP. Unfortunately, these attempts yielded ligand-bound structures, suggesting a small fraction of our GdmCl-treated protein retained prebound ligands. The refined model obtained in the presence of 0.5 mM L-glutamate contains residues 29–328, which equates to all of the 299 residues of mature VcGluP. VcGluP is composed of 2 α/β domains connected by a hinge composed of antiparallel β-sheets (residues 116–131 and 255–267). Domain 1 is composed of amino acids 29–115 and 268–328, and domain 2 consists of residues 132–254. Each domain contains three central parallel β-sheets bracketed by α-helices. Comparison with other SBP structures reveals that VcGluP belongs to Cluster F (Scheepers et al., 2016). In addition, each domain contains a disulfide bond; Cys53–Cys67 in domain 1 which connects a1 with B2, and Cys191–Cys216 in domain 2 that connects a8 and a9, although density for this disulfide is missing likely through radiation damage (Fig. 7 C). We also identified three Na^+^ ions bound to the surface of the protein (Fig. 7, A and B, purple spheres and Fig. S2), although due to their distance from the binding site, we predict that these are functionally irrelevant interactions and merely a consequence of the 2.4 M sodium malonate used during crystallization.

During refinement, we identified non-protein density in the cleft between the two domains, which we assigned to L-glutamate, which was present at 0.5 mM during crystallization (Fig. 7 and Fig. S2 A). As expected, in the presence of ligand, VcGluP adopts a closed conformation wherein the two domains completely envelope the ligand binding site (Fig. 7 B).

Analysis of the ligand binding site reveals that L-glutamate is coordinated by VcGluP via a hydrogen bonding network composed of direct mainchain and sidechain interactions, and indirectly through coordinated water molecules (Fig. 7, D and E). The interactions between the two domains of VcGluP are slightly asymmetric, with the majority of interactions coming from Domain 1 of VcGluP (Fig. 7 A). The α-carboxyl group of the bound L-glutamate makes sidechain and mainchain interactions with S75, mainchain interactions with G156 and hydrogen bonds to a water molecule, which is itself coordinated by the D157 sidechain. The amino group is coordinated by hydrogen bonding interactions with the side chains of Y45 (not shown in LigPlot+ representation), Q93 and E125, and the mainchain of M201. The side chain carboxyl of the ligand makes mainchain interactions with V44 and Y45, and hydrogen bonds to water molecules that are coordinated by S40, Y200, Y46, H123, G203, and E125. The positioning of Y45 and Y200 on either side of the binding site likely also provides additional hydrophobic interactions with the ligand.

### Biochemical characterization of binding site residue contributions

To investigate the binding determinants of VcGluP, we mutated the binding site residues Y45, S75, Q93, E125, Y200, and M201 to alanine, expressed and purified them, and assessed the impact of these mutations on ligand binding using DSF and tryptophan fluorescence. Each VcGluP variant was overproduced and purified, and their stability was initially assessed using DSF. While there was some variation in Tm under *apo* conditions for the different variants, the presence of a characteristic melt curve suggested they were all folded and stable (Fig. S3 A). We next used tryptophan fluorescence emission scans to screen for ligand binding by the mutants (Fig. S3 B). Addition of L-glutamate induced enhancement of fluorescence for all the mutants indicating that all of them can bind ligand. However, while maximum fluorescence enhancement could be achieved with only 50 μM L-glutamate or less for Y45A, S75A, E125A, Y200A, and M201A mutants, a higher concentration was required to saturate Q93A, indicating a diminished affinity for this mutant (Fig. S3 B).

Using the dose-responsive fluorescence change for each mutant, we determined their Kds for the primary ligand L-glutamate using tryptophan fluorescence. Our titrations revealed that all six mutants exhibited a reduced affinity for L-glutamate compared to wildtype (Table 1). The most deleterious mutations were Y45A and Q93A, which reduced affinity 213- and 990-fold, respectively, followed by S75A, which had a 14-fold lower affinity. E125A, M201A, and Y200A had relatively modest effects on affinity, reducing it by 1.3-, 2.1-, and 3.5-fold, respectively (Table 1).

**Table 1.** Equilibrium binding affinities for VcGluP wildtype and binding site mutants

Variant	Glu Kd (µM)	FC	*n*	Gln Kd (mM)	FC	*n*	Pyroglu Kd (mM)	FC	*n*
WT	0.065 ± 0.031	-	5	0.011 ± 0.002	-	5	0.050 ± 0.016	-	10
Y45A	13.88 ± 1.576	213	5	5.135 ± 0.864	466	6	2.000 ± 0.094	40	5
S75A	0.915 ± 0.126	14	5	0.073 ± 0.028	6.5	7	0.499 ± 0.050	10	5
Q93A	64.39 ± 13.74	990	10	ND	ND	5	18.62 ± 4.566	372	5
E125A	0.083 ± 0.023	1.3	5	0.025 ± 0.008	2.3	9	0.147 ± 0.010	2.9	6
Y200A	0.228 ± 0.088	3.5	6	0.022 ± 0.008	2	8	0.104 ± 0.022	2.1	3
M201A	0.139 ± 0.032	2.1	4	0.040 ± 0.009	3.6	9	0.302 ± 0.010	6	4

FC is the fold change in Kd compared to wildtype, *n* indicates the number of technical replicates performed, and error indicated is the standard deviation. Values are color coded to indicate the extent of Kd change for a mutant compared to wildtype (red being most deleterious, blue being a mild effect, and orange being middling).

We also used tryptophan fluorescence to determine the mutants’ affinities for L-glutamine and L-pyroglutamate and observed the same pattern with Y45A and Q93A reducing the affinity the most, and E125A, Y200A, and M201A having relatively little to no effect. Interestingly, the fold change in affinity is essentially the same for each mutant’s interaction with the three ligands, aside from Y45A where we observed a 466-fold change for L-glutamine, but only a 213-fold change for L-glutamate and a 40-fold change for L-pyroglutamate (Table 1).

### Modeling the VcGluP–VcGluQM interaction reveals a novel claw domain

**Figure 8.**
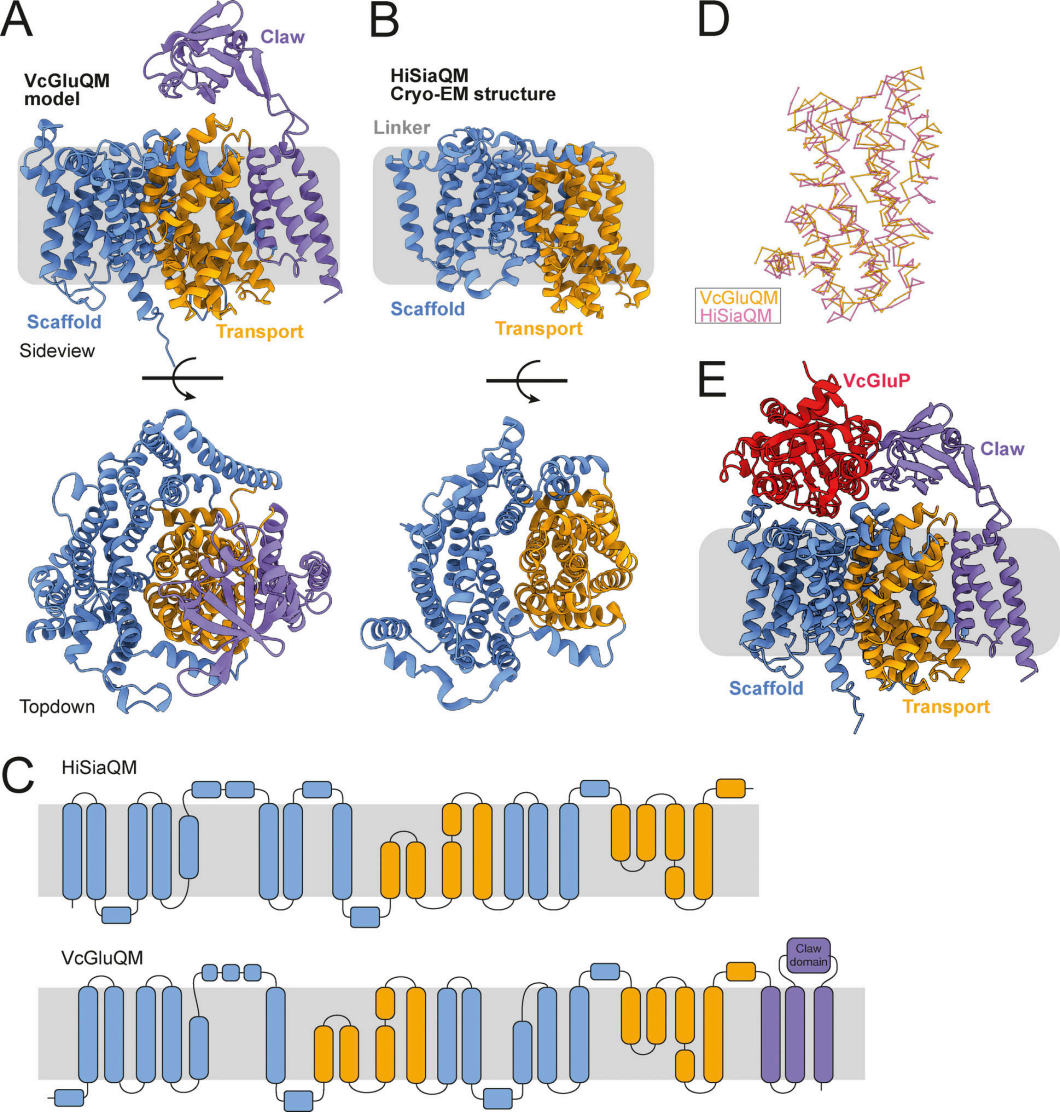
**Structural model of VcGluQM and the predicted interactions with VcGluP. (A)** Side view and top-down view of the structural model of VcGluQM generated by Alphafold2. The transport or “elevator” domain is colored orange, the scaffold (including the “Q” domain) is blue, and the claw domain is colored light purple. **(B)** Side view and top-down view of the cryo-EM structure of HiSiaQM (PDB ID: 7QE5), colored the same as for VcGluQM in A. **(C)** Topology maps of HiSiaQM and VcGluQM based on the cryo-EM structure and AlphaFold2 prediction, respectively. Maps colored as in A. **(D)** Superimposition of the chain traces of the transport domains of VcGluQM (orange) and HiSiaQM (pink). **(E)** Cartoon representation of the Colabfold model of the holocomplex VcGluPQM, colored as in A and with VcGluP colored red. VcGluQM and VcGluPQM Colabfold Models colored by pLDDT are presented in Fig. S4.

**Figure 9.**
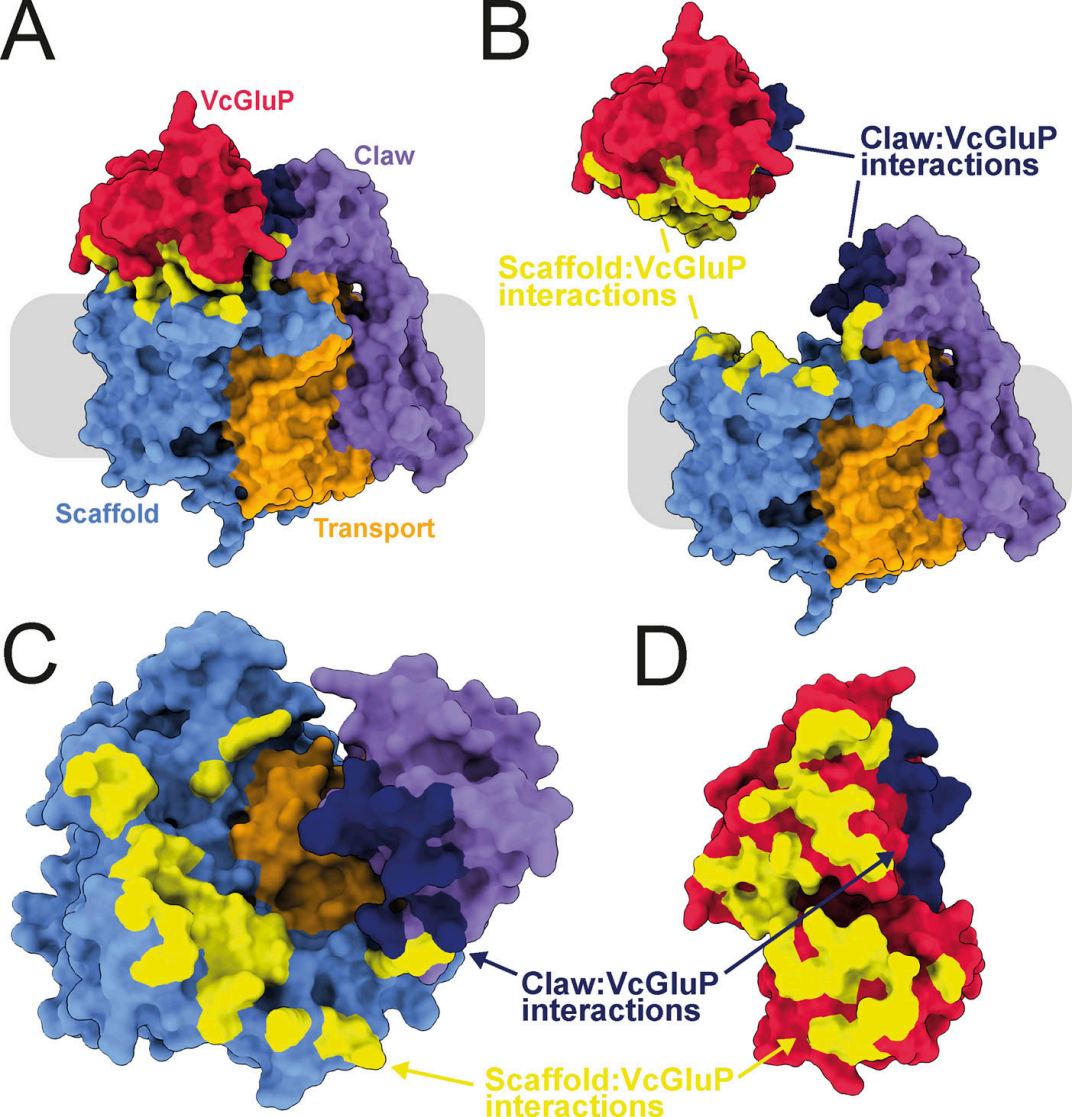
**Interfacial interactions between VcGluP and VcGluQM. (A)** Surface representation of VcGluPQM colored as in Fig. 8. Interactions between the scaffold domain and VcGluQM are in yellow and the interaction between VcGluP and the claw domain is in dark blue. **(B)** Same as A but the interface has been exploded. **(C and D)** Top-down view of VcGluQM showing interfacial contacts, and D bottom-up view of VcGluP showing interfacial contacts. Models colored by pLDDT are presented in Fig. S4.

There are currently no experimentally derived structures of TAXI membrane components, so to investigate the interaction between VcGluP with its cognate membrane component VcGluQM, we obtained the model of VcGluQM using Alphafold2 (Jumper et al., 2021). The model produced by Alphafold2 shared many core features identified in the cryo-EM structure of HiSiaQM (Currie et al., 2024; Peter et al., 2022), which is a membrane component from a DctP-type TRAP transporter. Both proteins consist of a transport or “elevator” domain and the clearly separable scaffold domain (otherwise known as a “stator” domain for TRAP transporters, Fig. 8, A and B; and Fig. S4). The VcGluQM model is in the inward-facing or “elevator down” state in which the predicted binding site is closer to the cytoplasmic side of the membrane. Most of the structural differences in these core domains appear to be localized to the scaffold domain, with the VcGluQM scaffold predicted to be arranged differently and slightly larger than the one in HiSiaQM (Fig. 8, A–C). However, there is substantial structural conservation in the transport domains, which superimpose very well with an RMSD of ∼4 Å over 230 residues (Fig. 8 D). These structural similarities may not be surprising because both proteins belong to the TRAP transporter family. However, they share a remarkably low amino acid sequence identity of 7.9% (alignment between full-length HiSiaQM and VcGluQM, and calculated using AlignMe [Stamm et al., 2014]). The amino acid sequences contributing to the transport domains, which are structurally conserved (Fig. 8 D), are only 14.1% identical. In addition to the low level of identity, VcGluQM is 221 amino acids longer than HiSiaQM, the majority of which comes from a 185 amino acid C-terminal extension in VcGluQM. This extension consists of three transmembrane helices attached to the transport domain and a globular domain belonging to the uncharacterized DUF3394 protein family (which we will refer to as the “claw” domain, Fig. 8). The claw domain is located in the periplasmic loop between the final two transmembrane helices, and is mainly composed of β-sheets (Fig. 8). The claw domain is predicted to make several polar and non-polar interactions with the transport domain of VcGluQM, but no interactions with the scaffold domain, and the positioning of the claw domain suggests that it comes into direct contact with VcGluP. To investigate this interaction, we modeled the holocomplex VcGluPQM using Colabfold (Mirdita et al., 2022), which revealed that, as with the models of HiSiaPQM (Peter et al., 2022), the SBP forms multiple interactions with the membrane component that is dominated by polar contacts (Fig. 9 and Fig. S4). However, unlike HiSiaP, which is predicted to interact with both the scaffold and transport domain (Peter et al., 2022), our model of VcGluPQM suggests that VcGluP does not directly interact with the transport domain of VcGluQM (Fig. 9). Instead, VcGluP forms multiple contacts with the C-terminal claw domain and scaffold domain with buried surface areas of 816.3 and 1342.4 Å^2^, respectively (calculated using the PDBePISA [Fig. 9] [Krissinel and Henrick, 2007]). Therefore, we predict that the claw domain plays a role in stabilizing the docking of the SBP with the membrane component (Fig. 9). As with the AlphaFold2 model of VcGluQM alone, the positioning of the transport domain suggests that VcGluQM is in its inward-facing state in the Colabfold model (Figs. 8 and 9).

When modeled independently using Alphafold2, VcGluP adopts a closed state identical to our crystal structure (Fig. S5 A). However, superimposition of our crystal structure of the closed, liganded VcGluP and the VcGluP structure predicted during the modeling of the holocomplex with Colabfold reveals that the ligand-free VcGluP in the holocomplex model adopts an open state (Fig. S5, B–D). With the caveat that these observations are based partially on structural models and require experimental validation, it is tempting to speculate that VcGluP’s open state observed in the Colabfold model is induced by interaction with the membrane component, and may represent a transitional stage of the transport cycle.

## Discussion

In this work, we have structurally characterized a TAXI TRAP SBP from the human pathogen *V. cholerae* and performed an in-depth analysis of the substrate binding determinants. We have shown that VcGluP preferentially binds glutamate and can also bind glutamine with a micromolar affinity. Glutamate uptake plays a key role in the osmoadaptability of this human pathogen and to the best of our knowledge, this is the only experimentally validated glutamate transporter identified in *V. cholerae*. We have demonstrated that ligand binding involves interactions with side chains and an extensive water network. In addition, we have shown that glutamine and tyrosine are *the* critical ligand binding residues, and VcGluP lacks a functionally important binding site arginine residue, which is a hallmark of DctP-type TRAP SBPs. Analysis of Alphafold2-based models of the membrane component suggests a unique arrangement hitherto unseen for TRAP transporters, in which a C-terminal extension forms a globular periplasmic domain and makes numerous interactions with the SBP.

This work is the first to investigate the functional contributions of binding site residues in a TAXI TRAP transporter SBP. Characterization of the substrate range and selectivity of DctP-type TRAP transporter SBPs has revealed that they can bind to a diverse range of compounds, the majority of which contain at least one carboxyl group (Mulligan et al., 2011; Vetting et al., 2015). The numerous structures of DctP-type SBPs have revealed that the carboxyl group of the ligand is coordinated by an arginine sidechain, which is highly conserved in DctP-type SBPs and serves as a selectivity filter for binding (Fischer et al., 2015). Our structure and binding site characterization of VcGluP has revealed that no arginine (or lysine) plays a role in the substrate binding site, revealing a key difference between the two TRAP sub-families.

Binding of L-glutamate by VcGluP is facilitated by a range of sidechain interactions (Fig. 7). In addition, an extensive water network also provides several key interactions (Fig. 7, D and E), which is a feature observed previously in other TRAP SBP binding sites (Darby et al., 2019). From our structure, we identified six residues potentially involved in ligand binding; Y45, S75, Q93, E125, Y200, and M201. However, we found that binding affinity was only severely diminished when Y45 and Q93 were mutated to alanine, reducing the affinity by 213- and 990-fold (Table 1), respectively, suggesting that these residues are the key binding determinants for VcGluP.

Comparison of VcGluP’s binding site reveals that it is almost identical to the binding site of the glutamate/glutamine TAXI, TtGluBP from *T. thermophilus* (TTHA1157, Fig. S6 B) (Takahashi et al., 2004). While both proteins have the same structural organization (categorized as Cluster F SBPs in the structure-based SBP classification system [Scheepers et al., 2016]) and superimpose with an RMSD of 2.1 Å (Fig. S6 A), the identical binding site was unexpected considering they did not coalesce on our phylogenetic tree and are only 38% identical to each other (Fig. 1). The positioning of the key residue Y45 is identical for TtGluBP and VcGluP (Fig. S6, B and C). However, while Gln93 in VcGluP and its equivalent in TtGluBP, Gln78, take up identical positioning, Gln93 in VcGluP directly interacts with the amino group of the ligand, whereas, Gln78 in TtGluBP does not. Instead, Thr187 (which is in the equivalent position to M201 in VcGluP), interacts with the amino group, relegating Gln87 to contributing only hydrophobic interactions to the binding site (Fig. S6 C). Based on this, we predict that Gln78 may not play as crucial a role in ligand binding in TtGluBP as it does in VcGluP. A feature of both TtGluBP and VcGluP is the positioning of two tyrosine residues on either side of the glutamate (Y45 and Y200 in VcGluP) (Takahashi et al., 2004). BugE, from the tripartite tricarboxylate transporter (TTT) family of binding protein-dependent secondary active transporters, also binds glutamate and exhibits the same overall architecture all SBPs share (Huvent et al., 2006). While there is no sequence similarity between the VcGluP and BugE, and the overall binding site composition is different, both proteins use aromatic residues (Phe in BugE and Tyr in VcGluP) to sandwich the aliphatic chain of the glutamate providing hydrophobic interactions (Fig. 7 D) (Huvent et al., 2006). Aromatic residues are also prominent in the binding site of the pyroglutamate-specific DctP-type TRAP transporters from *Bordetella pertussis*, Dct6 and Dct7, but in these proteins the aromatic residues are a combination of tryptophan and tyrosine (Rucktooa et al., 2007). To support the importance of these aromatic residues, the equivalent residue to Y45 is found in 78% of the TAXI sequences in our alignment (Data S1). The equivalent residue to Y200 is only found in 20% of sequences reflective of its negligible contribution to binding (Table 1). The residue that appeared to contribute most significantly to binding, Q93, is found in 54% of the sequences in our alignment (Data S1), suggesting that this is possibly a variable site that will change and allow different TAXIs to accommodate different ligands. However, more information on the range of ligands and the binding determinants of TAXI SBPs is required to evaluate this.

The cryo-EM structures of DctP-type TRAP membrane components have revealed important insight into the transport mechanism of TRAP transporters and have confirmed previous predictions (Davies et al., 2023; Peter et al., 2022; Ovchinnikov et al., 2014). We currently lack any experimental structures of TAXI-TRAP membrane components. While we show here that they are predicted to largely resemble DctP-type TRAP membrane components, the low sequence identity between DctP- and TAXI-type membrane components will likely result in mechanistic differences. Indeed, significant divergence has also been observed by the TAXI-TRAP from *P. mirabilis* using an H^+^ gradient, unlike the Na^+^-driven DctP-type TRAP transporters that have been characterized (Roden et al., 2023). Investigation into the predicted structure of VcGluQM revealed that it is substantially larger than the structurally characterized DctP-type TRAP membrane components (Fig. 8). Most of the size difference between VcGluQM and HiSiaQM is accounted for by VcGluQM’s C-terminal extension that contains three TM helices and a periplasmic globular domain that we predict to interacts with the SBP (Fig. 9). This extension has not been reported for any DctP-type TRAP transporters, suggesting that it is a TAXI-specific addition, and it is highly reminiscent of the similar domain in the maltose ABC transporter, MalEFGK_2_ (Oldham et al., 2007). However, the claw domain does not appear to be a general feature of TAXI TRAP membrane components either. Structural comparison of the *P. mirabilis* TAXI membrane component PMI1055 and VcGluQM reveals that, while both proteins have the three TM C-terminal extension, only VcGluQM has the globular claw domain (Fig. S7). Analysis of the Alphafold2-derived models of the membrane components associated with the 59 TAXI transporters in our phylogenetic tree reveals that only the TAXI TRAP transporters in the vicinity of VcGluP are predicted to have this claw domain. All other TAXI transporters in our tree, if they have an associated membrane component gene, lack this claw domain in their membrane components.

The function of the claw domain is currently unknown, but one possibility is that it enhances the affinity between the SBP and membrane component. Recently, analysis of the TAXI family revealed that a large proportion of TAXI SBPs are lipoproteins that are fused via a cysteine to a lipid headgroup (Roden et al., 2023). This membrane tethering would substantially increase the local SBP concentration and overcome a low-affinity interaction between SBP and membrane component. Analysis of VcGluP’s sequence using SignalP 6.0 reveals that it is predicted with 99.9% confidence to contain a Sec/SPI signal peptide (Teufel et al., 2022), meaning that VcGluP would move unrestricted in the periplasm rather than being tethered to the membrane. Therefore, in this case, the claw domain may simply be stabilizing the interaction between the membrane component and an untethered SBP. However, further investigation into the spread of the claw domain and how it correlates with membrane tethering is required. Finally, while our structural and biochemical analyses of VcGluP suggest very strongly that VcGluPQM is a glutamate transporter, to demonstrate this conclusively, transport activity would need to be measured in vitro or in vivo. This future work is also required to investigate whether glutamine and pyroglutamate are transportable substrates of this transporter, or just able to bind to VcGluP, in which case, they may act as transport inhibitors.

In this work, we have shown that VcGluPQM is likely an L-glutamate transporter from the Gram-negative, halophilic intestinal pathogen *V*.* cholerae* which still poses a significant threat to human life, especially in lower income countries (Ali et al., 2015). As *V. cholerae* naturally inhabits estuarine environments, it undergoes substantial fluctuations in the ionic strength of the growth media during its lifecycle, which requires a robust response to avoid cell lysis. While glutamate is itself a known osmolyte (Csonka, 1989), and its uptake by *V. cholerae* increases under elevated osmotic pressure (Munro and Gauthier, 1994), it is actually used as a precursor for ectoine, which is one of the main compatible solutes used by *V. cholerae* (Pflughoeft et al., 2003). Therefore, glutamate uptake via VcGluPQM likely contributes substantially to osmoadaptability allowing the pathogen to survive in elevated external osmolarity during niche adaptation and in the intestine during infection (Pflughoeft et al., 2003).

Here, we have characterized VcGluP, a TAXI TRAP SBP from *V. cholerae*, the first experimentally validated glutamate transporter identified in this pathogen. This work has shed new light on the binding determinants of TAXI transporters, provided new insight into the protein:protein interactions of binding protein-dependent transporters, and has identified a transporter in a human pathogen that likely influences its environmental adaptability.

## Supplementary Material

Table S1 is a list of *E. coli* metabolites between 144 and 148 Da.

Table S2 contains the full datasets from DSF binding analysis presented in Fig. 3 and Fig. 5.

Table S3 shows data collection and refinement statistics.

Data S1 is a multiple sequence alignment of a variety of TAXI binding proteins.

SourceData F3 is the source file for Fig. 3.

## Data Availability

The data underlying all figures are available in the article and its online supplemental material. The data underlying the X-ray structures have been deposited in the Protein Data Bank. PDB ID: 8S4J.
