# Supplementary material for: Structure and selectivity of a glutamate-specific TAXI TRAP binding protein from Vibrio cholerae
Source: J Gen Physiol. 2024 Nov 18;156(12):e202413584. doi: 10.1085/jgp.202413584 (PMC11574862; doi:10.1085/jgp.202413584)
Supplement: Table S1 — is a list of E. coli metabolites between 144 and 148 Da. [file JGP_202413584_TableS1.docx]

**Supplementary table 1.** List of E. coli metabolites between 144-148 Da. Data obtained from EcoCyc.org.

| **Compound Name** | **Mol. Wt.** |
| --- | --- |
| 2-oxoglutarate | 144.084 |
| 5,6-dihydroxyuracil | 144.087 |
| 2-oxoglutaramate | 144.107 |
| adipate | 144.127 |
| keto-ω-methylpantoyl lactone | 144.127 |
| 5-hydroxy-6-hydrothymine | 144.13 |
| ethyl-2-methylacetoacetate | 144.17 |
| dehydrolysine | 144.173 |
| N-methyl-DL-aspartate | 145.115 |
| (S)-2-aceto-2-hydroxybutanoate | 145.135 |
| 2-dehydropantoate | 145.135 |
| 3-hydroxy-3-methyl-2-oxopentanoate | 145.135 |
| 4-hydroxy-2-oxohexanoate | 145.135 |
| 5-hydroxypipecolate | 145.158 |
| 8-hydroxyquinoline | 145.16 |
| (S)-4-amino-4,5-dihydro-2-thiophenecarboxylate | 145.176 |
| γ-butyrobetaine | 145.201 |
| (3R)-oxaloglycolate | 146.056 |
| (R)-2-hydroxyglutarate | 146.099 |
| (R)-citramalate | 146.099 |
| (S)-2-hydroxyglutarate | 146.099 |
| (S)-citramalate | 146.099 |
| (Z)-3-ureidoacrylate peracid | 146.102 |
| uracil glycol | 146.102 |
| (2S, 3S)-3-methylaspartate | 146.122 |
| 2-methylaspartate | 146.122 |
| D-glutamate | 146.122 |
| L-glutamate | 146.122 |
| N-hydroxy-N-isopropyloxamate | 146.122 |
| D-galactal | 146.143 |
| a coumarin | 146.145 |
| D-glutamine | 146.146 |
| glycylsarcosine | 146.146 |
| L-alanyl-glycine | 146.146 |
| L-glutamine | 146.146 |
| ethyl-(2R)-methyl-(3S)-hydroxybutanoate | 146.186 |
| 5,6-dimethylbenzimidazole | 146.191 |
| tetraethylammonium | 146.295 |
| calcium chloride dihydrate | 147.016 |
| phosphonocytosine | 147.05 |
| N-oxalylglycine | 147.087 |
| 2-dehydro-3-deoxy-D-pentonate | 147.107 |
| O-acetyl-L-serine | 147.13 |
| (R)-2,3-dihydroxy-3-methylpentanoate | 147.15 |
| (R)-pantoate | 147.15 |
| cinnamate | 147.153 |
| 4-(methylsulfanyl)-2-oxobutanoate | 147.168 |
| (R)-β-lysine | 147.197 |
| 2,5-diaminohexanoate | 147.197 |
| D-lysine | 147.197 |
| L-lysine | 147.197 |
