# Supplementary material for: Structure and selectivity of a glutamate-specific TAXI TRAP binding protein from Vibrio cholerae
Source: J Gen Physiol. 2024 Nov 18;156(12):e202413584. doi: 10.1085/jgp.202413584 (PMC11574862; doi:10.1085/jgp.202413584)
Supplement: Table S2 — contains the full datasets from DSF binding analysis presented in Fig. 3 and Fig. 5. [file JGP_202413584_TableS2.docx]

**Supplementary Table 2. Full datasets from DSF binding analysis presented in Figures 3 and 5.**

|  | **Sample** | **Tm Replicate 1 (°C)** | **Tm Replicate 2 (°C)** | **Tm Replicate 3 (°C)** | **Average Tm (°C)** | **Standard Deviation** |
| --- | --- | --- | --- | --- | --- | --- |
| Fig. 3C | Apo | 46.73 | 47.72 | 45.74 | 46.73 | 0.8109 |
|  | 1 mM L-Glu | 55.67 | 54.68 | 55.67 | 55.34 | 0.4683 |
|  | 1 mM L-Gln | 49.71 | 50.71 | 49.71 | 50.04 | 0.4686 |
|  | 1 mM L-Lys | 43.75 | 44.74 | 44.74 | 44.41 | 0.4676 |
|  | 1 mM L-Asp | 47.72 | 45.74 | 47.73 | 47.06 | 0.9369 |
|  | 1 mM α-KG | 50.71 | 45.74 | 45.74 | 47.39 | 2.3424 |
|  | 1 mM Adipate | 43.75 | 45.74 | 44.74 | 44.74 | 0.8112 |
| Fig. 5A | Apo | 55.67 | 55.67 | 55.67 | 55.67 | 0 |
|  | 1 mM L-Glu | 71.57 | 71.57 | 71.57 | 71.57 | 0.0001226 |
|  | 1 mM D-Glu | 58.65 | 58.65 | 58.65 | 58.65 | 0.0005138 |
| Fig. 5B | Apo | 46.73 | 46.73 | 46.74 | 46.73 | 0.002640 |
|  | 1 mM L-Gln | 49.71 | 50.70 | 50.70 | 50.37 | 0.4685 |
|  | 5 mM L-Gln | 53.68 | 53.68 | 53.68 | 53.68 | 0.0002985 |
|  | 15 mM L-Gln | 55.67 | 55.67 | 55.67 | 55.67 | 3.597E-06 |
|  | 1 mM D-Gln | 46.73 | 45.74 | 46.73 | 46.40 | 0.4682 |
|  | 5 mM D-Gln | 46.72 | 47.72 | 46.73 | 47.06 | 0.4684 |
|  | 15 mM D-Gln | 48.72 | 48.72 | 48.72 | 48.72 | 0.0002554 |
