# Supplementary material for: Structure and selectivity of a glutamate-specific TAXI TRAP binding protein from Vibrio cholerae
Source: J Gen Physiol. 2024 Nov 18;156(12):e202413584. doi: 10.1085/jgp.202413584 (PMC11574862; doi:10.1085/jgp.202413584)
Supplement: Table S3 — shows data collection and refinement statistics. [file JGP_202413584_TableS3.docx]

**Supplementary Table 3. Data collection and refinement statistics.**

Statistics for the highest-resolution shell are shown in parentheses.

|  | **Vc0430 Bound to L-Glutamate** |
| --- | --- |
| **Wavelength** |  |
| **Resolution range** | 69.64 - 1.7 (1.761 - 1.7) |
| **Space group** | R 3 2 :H |
| **Unit cell** | 126.66 126.66 180.28 90 90 120 |
| **Total reflections** | 1242043 (122113) |
| **Unique reflections** | 61144 (6061) |
| **Multiplicity** | 20.3 (20.1) |
| **Completeness (%)** | 99.99 (99.98) |
| **Mean I/sigma (I)** | 26.31 (3.42) |
| **Wilson B-factor** | 27.60 |
| **R-merge** | 0.06415 (0.9057) |
| **R-meas** | 0.0658 (0.9292) |
| **R-pim** | 0.01456 (0.2063) |
| **CC1/2** | 1 (0.917) |
| **CC*** | 1 (0.978) |
| **Reflections used in refinement** | 61140 (6066) |
| **Reflections used for R-free** | 3035 (317) |
| **R-work** | 0.1902 (0.3618) |
| **R-free** | 0.2136 (0.3516) |
| **CC (work)** | 0.969 (0.824) |
| **CC (free)** | 0.964 (0.809) |
| **Number of non-hydrogen atoms** | 2698 |
| **macromolecules** | 2425 |
| **ligands** | 15 |
| **solvent** | 258 |
| **Protein residues** | 300 |
| **RMS(bonds)** | 0.007 |
| **RMS(angles)** | 0.96 |
| **Ramachandran favored (%)** | 96.97 |
| **Ramachandran allowed (%)** | 3.03 |
| **Ramachandran outliers (%)** | 0.00 |
| **Rotamer outliers (%)** | 1.52 |
| **Clashscore** | 1.65 |
| **Average B-factor** | 28.09 |
| **macromolecules** | 26.92 |
| **ligands** | 27.41 |
| **solvent** | 39.12 |
