# Supplementary material for: Structure and selectivity of a glutamate-specific TAXI TRAP binding protein from Vibrio cholerae
Source: J Gen Physiol. 2024 Nov 18;156(12):e202413584. doi: 10.1085/jgp.202413584 (PMC11574862; doi:10.1085/jgp.202413584)
Supplement: Data S1 — is a multiple sequence alignment of a variety of TAXI binding proteins. [file JGP_202413584_DataS1.pdf]

Data S1. Multiple sequence alignment of 59 TAXI SBPs. Sequence alignment to accompany the cladogram in Figure 1. Protein names are colour coded to match the tree in Figure 1 and the binding site residues mutated to alanine are indicated with an arrow.

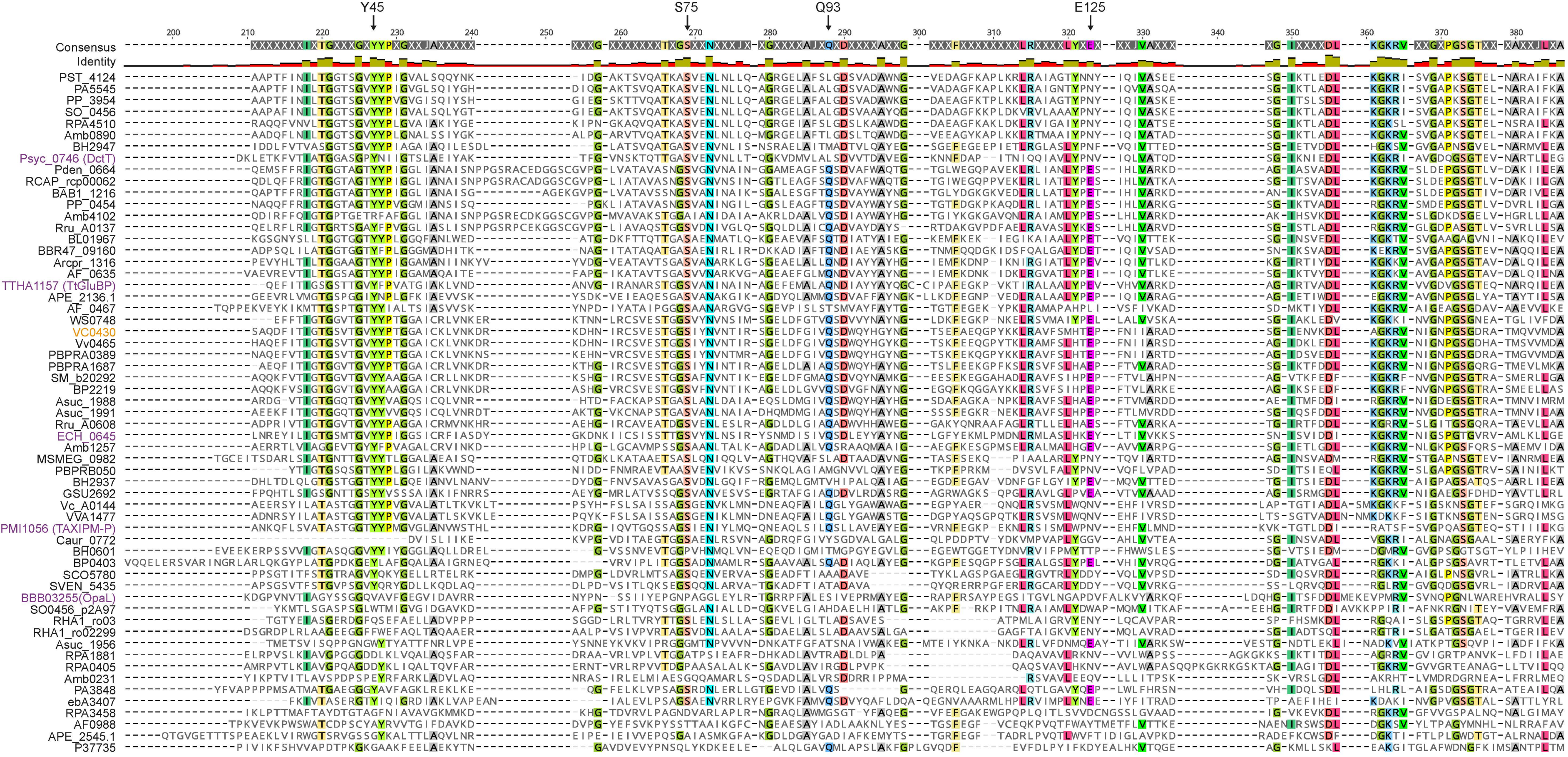

Y45
S75
Q93
E125
200
210
220
230
240
250
260
270
280
290
300
310
320
330
340
350
360
370
380
Consensus
Identity
PST_4124
PA5545
PP_3954
SO_0456
RPA4510
Amb0890
BH2947
Psyc_0746 (DctT)
Pden_0664
RCAP_rcp00062
BAB1_1216
PP_0454
Amb4102
Rru_A0137
BL01967
BBR47_09160
Arcpr_1316
AF_0635
TTHA1157 (TtGluBP)
APE_2136.1
AF_0467
WS0748
VC0430
Vv0465
PBPRA0389
PBPRA1687
SM_b20292
BP2219
Asuc_1988
Asuc_1991
Rru_A0608
ECH_0645
Amb1257
MSMEG_0982
PBPRB050
BH2937
GSU2692
Vc_A0144
VVA1477
PMI1056 (TAXIPM-P)
Caur_0772
BH0601
BP0403
SCO5780
SVEN_5435
BBB03255(OpaL)
SO0456_p2A97
RHA1_ro03
RHA1_ro02299
Asuc_1956
RPA1881
RPA0405
Amb0231
PA3848
ebA3407
RPA3458
AF0988
APE_2545.1
P37735

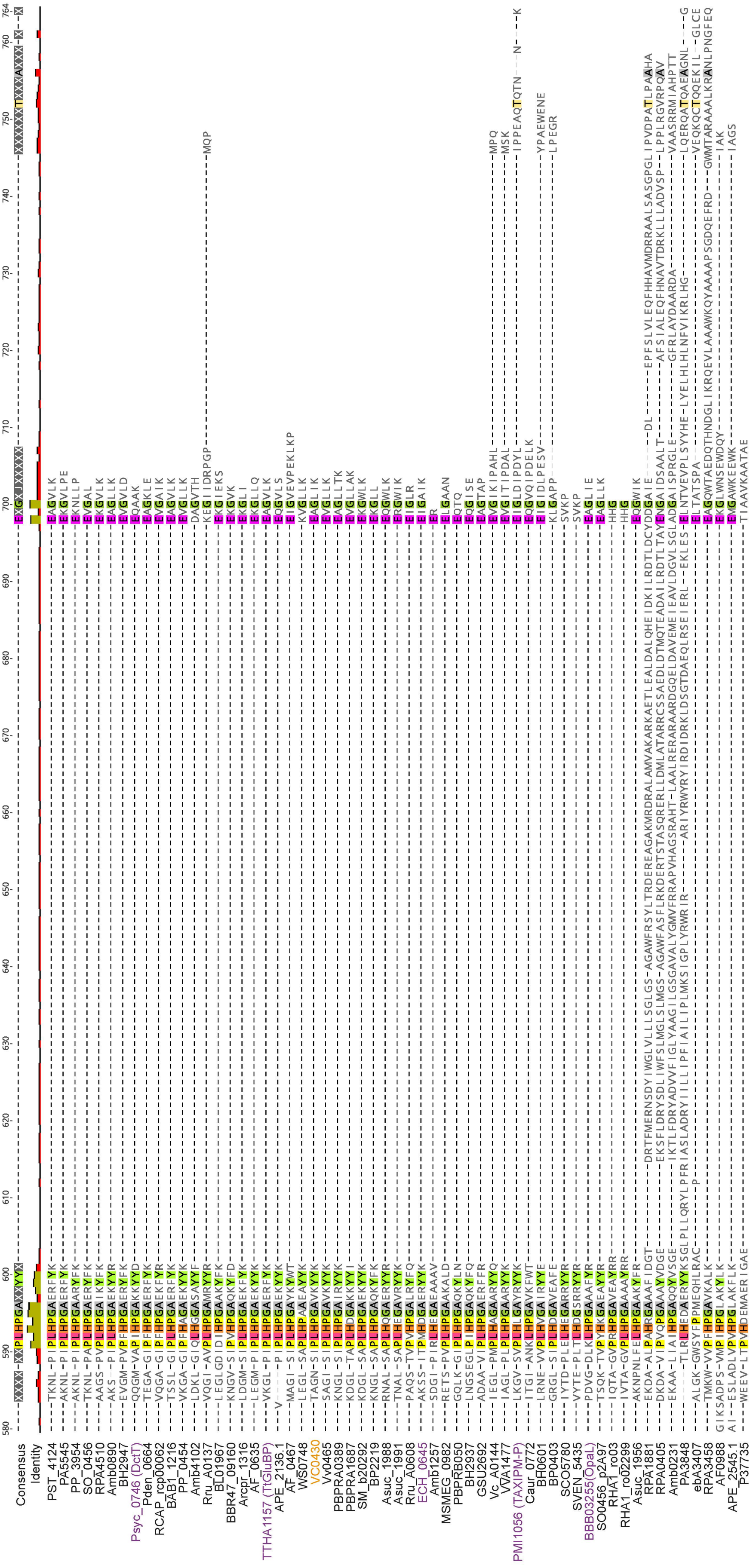

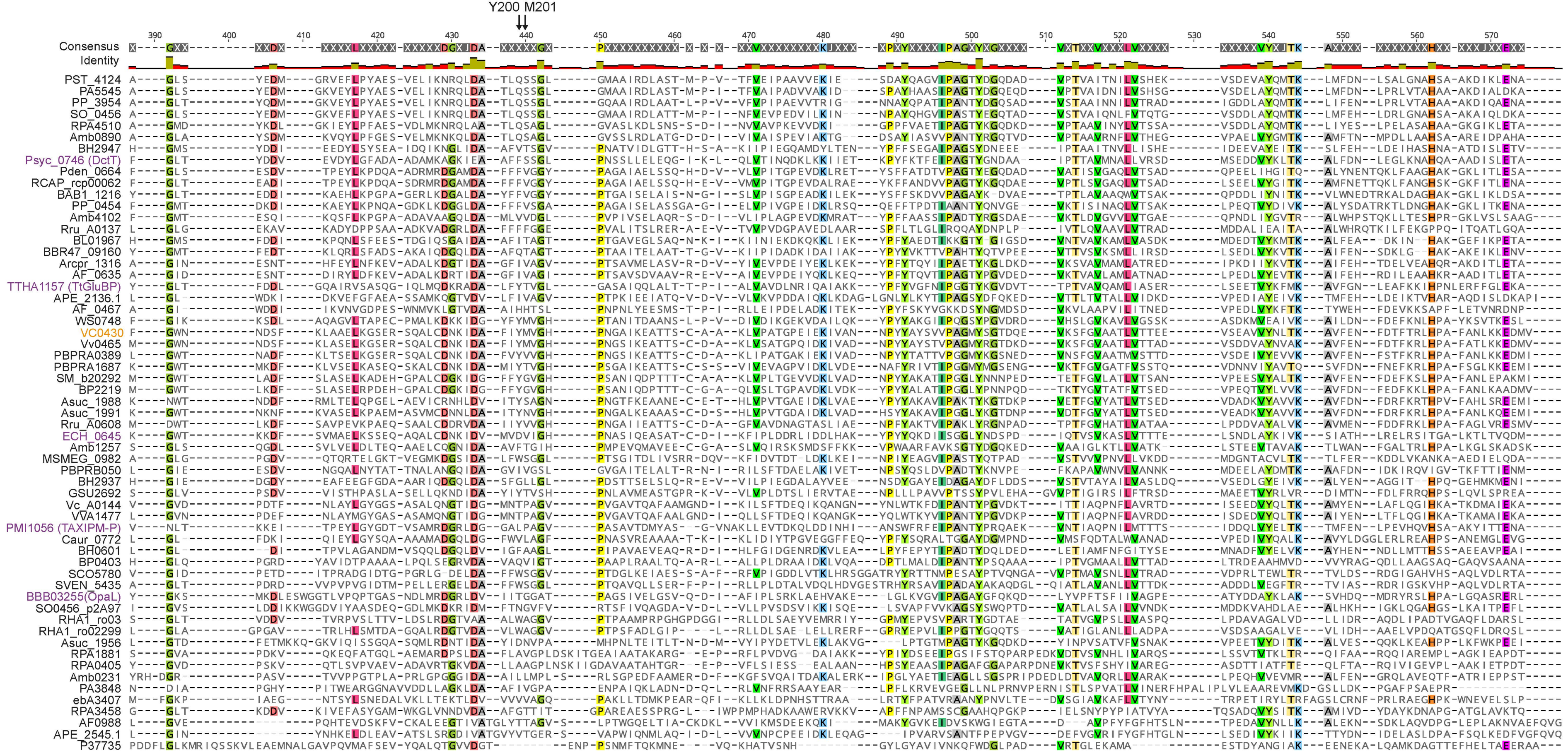
Y200 M201
Consensus
Identity
PST_4124
PA5545
PP_3954
SO_0456
RPA4510
Amb0890
BH2947
Psyc_0746 (DctT)
Pden_0664
RCAP_rcp00062
BAB1_1216
PP_0454
Amb4102
Rru_A0137
BL01967
BBR47_09160
Arcpr_1316
AF_0635
TTHA1157 (TtGluBP)
APE_2136.1
AF_0467
WS0748
VC0430
Vv0465
PBPRA0389
PBPRA1687
SM_b20292
BP2219
Asuc_1988
Asuc_1991
Rru_A0608
ECH_0645
Amb1257
MSMEG_0982
PBPRB050
BH2937
GSU2692
Vc_A0144
VVA1477
PMI1056 (TAXIPM-P)
Caur_0772
BH0601
BP0403
SCO5780
SVEN_5435
BBB03255(OpaL)
SO0456_p2A97
RHA1_ro03
RHA1_ro02299
Asuc_1956
RPA1881
RPA0405
Amb0231
PA3848
ebA3407
RPA3458
AF0988
APE_2545.1
P37735

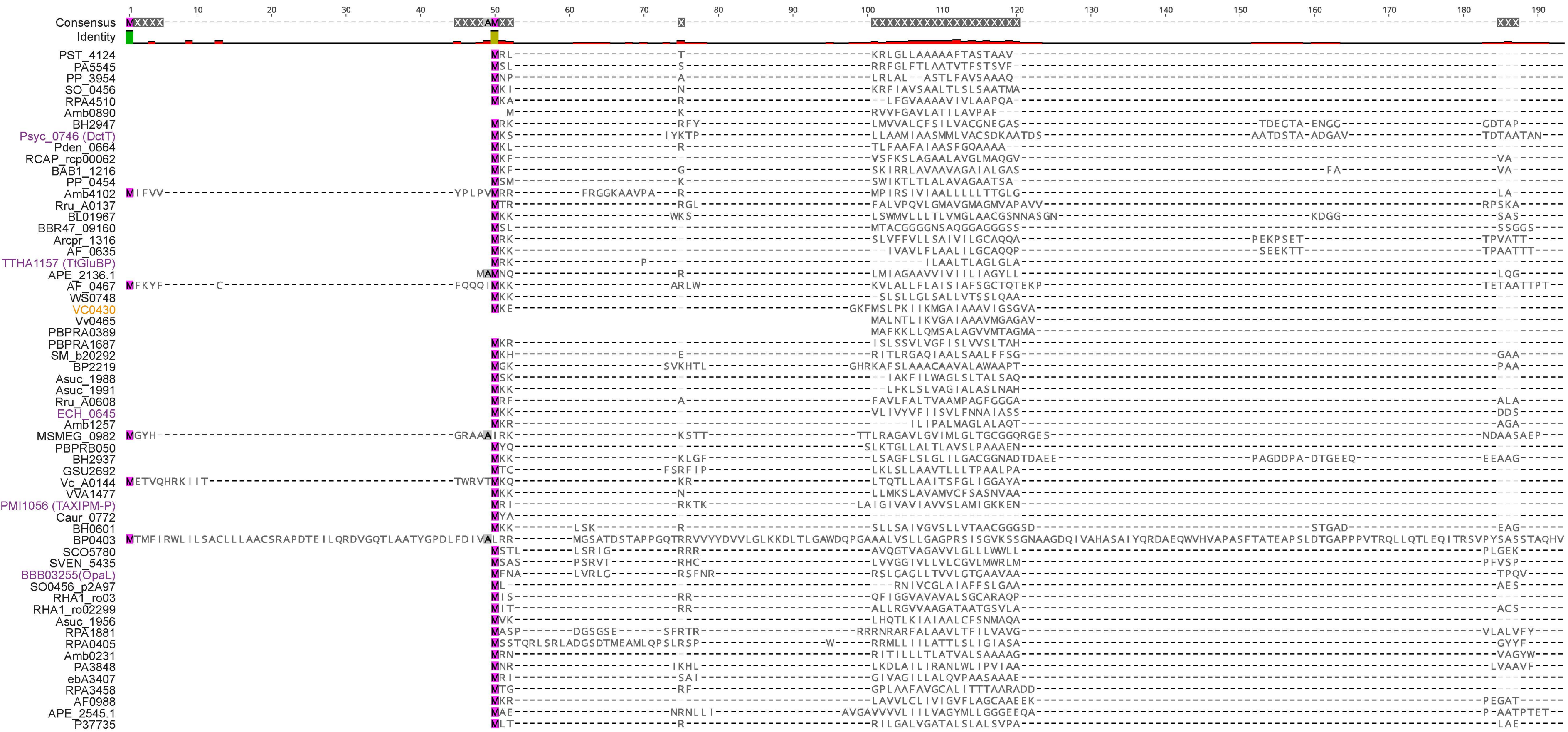
Consensus
Identity
PST_4124
PA5545
PP_3954
SO_0456
RPA4510
Amb0890
BH2947
Psyc_0746 (DctT)
Pden_0664
RCAP_rcp00062
BAB1_1216
PP_0454
Amb4102
Rru_A0137
BL01967
BBR47_09160
Arcpr_1316
AF_0635
TTHA1157 (TtGluBP)
APE_2136.1
AF_0467
WS0748
VC0430
Vv0465
PBPRA0389
PBPRA1687
SM_b20292
BP2219
Asuc_1988
Asuc_1991
Rru_A0608
ECH_0645
Amb1257
MSMEG_0982
PBPRB050
BH2937
GSU2692
Vc_A0144
VVA1477
PMI1056 (TAXIPM-P)
Caur_0772
BH0601
BP0403
SCO5780
SVEN_5435
BBB03255(OpaL)
SO0456_p2A97
RHA1_ro03
RHA1_ro02299
Asuc_1956
RPA1881
RPA0405
Amb0231
PA3848
ebA3407
RPA3458
AF0988
APE_2545.1
P37735
