# Supplementary figures and images for: Structure and selectivity of a glutamate-specific TAXI TRAP binding protein from Vibrio cholerae
Source: J Gen Physiol. 2024 Nov 18;156(12):e202413584. doi: 10.1085/jgp.202413584 (PMC11574862; doi:10.1085/jgp.202413584)

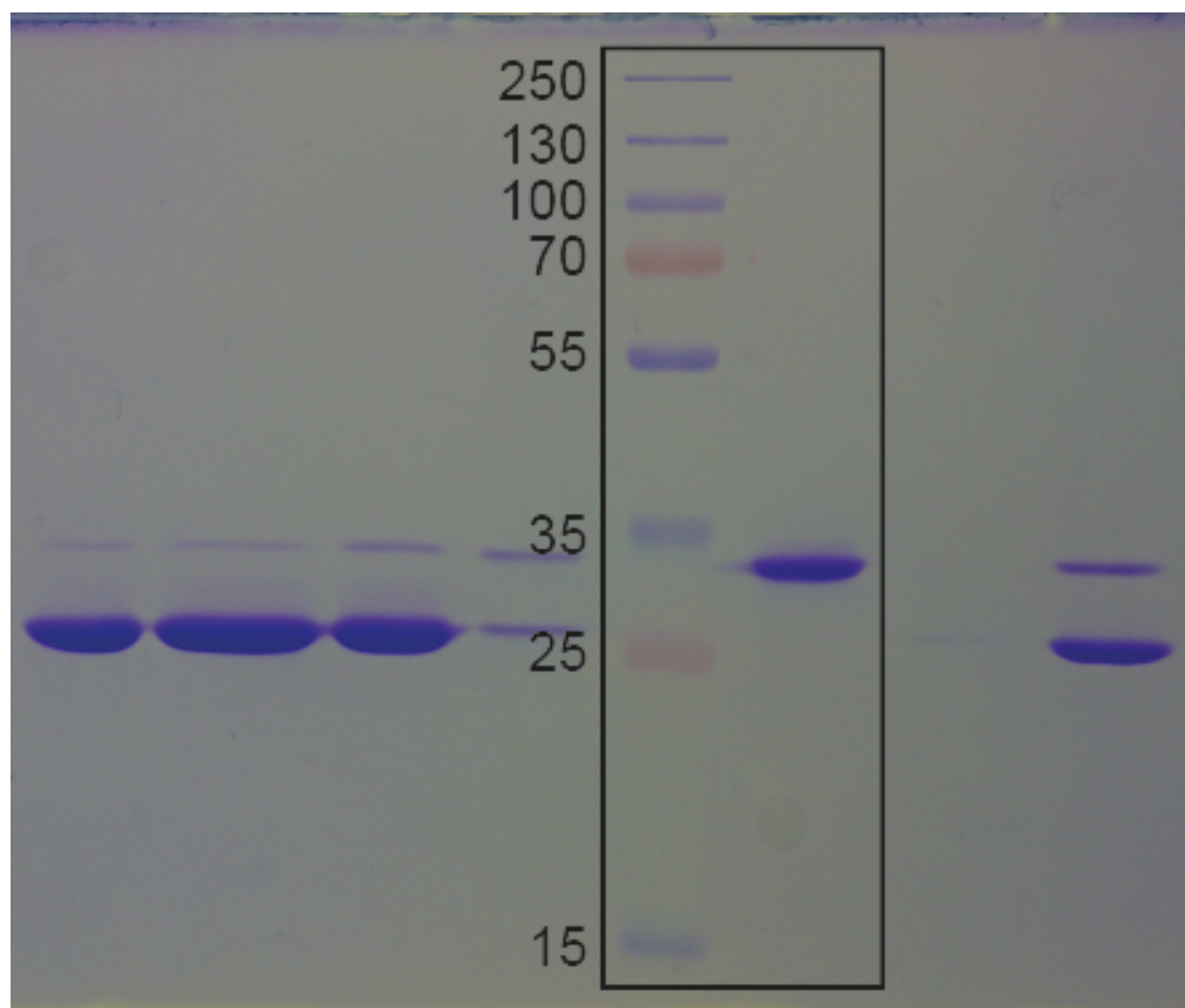

Supplement: SourceData F3 — is the source file for Fig. 3. [file JGP_202413584_SourceDataF3.pdf]
